# Early Occupational Therapy Intervention post-stroke (EOTIPS): A randomized controlled trial

**DOI:** 10.1371/journal.pone.0308800

**Published:** 2024-08-19

**Authors:** Patricia García-Pérez, María Carmen Rodríguez-Martínez, Alejandro Gallardo-Tur, Encarnación Blanco-Reina, Carlos de la Cruz-Cosme, José Pablo Lara

**Affiliations:** 1 Physiology Department, Faculty of Medicine, University of Malaga, Malaga, Spain; 2 Occupational Therapy Department, Hospital Civil, Malaga, Spain; 3 Department of Physiotherapy, Faculty of Health Sciences, University of Malaga, Malaga, Spain; 4 Brain Health Unit, Centro de Investigaciones Médico-Sanitarias (CIMES), Malaga, Spain; 5 Biomedical Research Institute of Malaga-Nanomedicine Platform (IBIMA-BIONAND Platform), Malaga, Spain; 6 Neurology Department, Virgen de la Victoria University Hospital, Malaga, Spain; 7 Pharmacology and Therapeutics Department, Faculty of Medicine, University of Malaga, Malaga, Spain; 8 Medicine and Dermatology Departments, Faculty of Medicine, University of Malaga, Malaga, Spain; Transilvania University of Brasov: Universitatea Transilvania din Brasov, ROMANIA

## Abstract

**Background:**

Occupational therapy (OT) is an effective evidence-based intervention that positively influences stroke patients’independence recovery, leading to new opportunities for better quality of life outcomes.

**Objectives:**

To explore the effectiveness of an early OT intervention program (EOTIPS) in the process of hospital to home discharge after stroke in Spain.

**Material and methods:**

We conducted a prospective, randomized controlled clinical trial that included 60 adults who suffered a stroke and were discharged home. Participants assigned to the experimental group (n = 30) were included in EOTIPS and compared with a control group (n = 30). Evaluations assessed quality of life (Stroke and Aphasia Quality of Life Scale [SAQOL-39]), functional independence (Modified Rankin Scale [mRS], Barthel Index [BI] and Stroke Impact Scale-16 [SIS-16]), perceptual-cognitive skills (Montreal Cognitive Assessment [MoCA]), upper limb function (Fugl Meyer Assessment [FMA]), mobility (Berg Balance Scale [BBS] and Timed Up & Go [TUG]), communication skills (Communicative Activity Log [CAL]) and mood disorders (Beck Depression Inventory–II [BDI-II] and Hamilton Anxiety Scale [HAM-A]); they were completed within two weeks post-stroke and after three months follow-up. Statistical analysis included intent-to-treat analysis, considering all participants (dropouts as failures), and efficacy analysis, considering only end-of-treatment participants.

**Results:**

Participants in the intervention group showed a significant better evolution in the main outcome measure of quality of life (SAQOL-39 *p* = .029), as well as for independence (mRS*p* = .004), perceptual-cognitive skills (MoCA *p* = .012)and symptoms of depression (BDI-II *p* = .011) compared to the control group.

**Conclusions:**

EOTIPS was effective in improving quality of life, as well as enhancing perceptual-cognitive skills, independence and reducing levels of depression for patients who suffered a stroke in a Spanish cohort and could be considered as an applicable non-pharmacologic therapeutic tool that can lead to patients’ positive outcomes after stroke.

This study was registered on ClinicalTrials.gov with the identifier NCT04835363.

## Introduction

Stroke is a significant health, social, and economic problem that represents the first cause of adult disability in Europe. However, advances in stroke prevention and treatment suppose that more people survive and live with its long-term consequences [1]. In addition, the number of younger persons suffering a stroke is increasing, resulting in a long-term health problem, affecting social, community, work and leisure activities. The presence of depressive symptoms, cognitive impairment, functioning, mobility and daily life activities limitations are most frequently and consistently associated with poor participation outcomes and a lower quality of life [2].

Initially, a systematic review was carried out in order to study interventions within the scope of Occupational Therapy (OT) to support discharge from an acute care hospital to home. According to the results, an early OT intervention can be effective in terms of functional recovery and can lead to caregiver’s self-efficacy. OT practitioners working with adults who suffered a stroke must understand the implications of their clients’ limitations on occupational performance and choose individualized interventions based on clinical reasoning and scientific evidence. The included studies agreed that individual planning and rehabilitation should start before discharge and patients with both social and physical needs could benefit from multidisciplinary services [3–5], which could lead to a more rapid independence recovery [6].

After stroke, patients may experience impaired motor and cognitive impairment, difficulties performing activities, and reduced health-related quality of life, which can place a burden on caregivers and society [7]. Early mobilization and home-based rehabilitation reduce disability and increases quality of life and it has been shown to be more cost-effective when compared to standard care [4, 8]. Patients should be informed and aware of the importance of discharge transition and carefully plan this moment together with professionals and caregivers, since the impact of this situation can affect quality of life and functionality [9]. OT plays an important role in a multidisciplinary approach to the treatment of cognitive and motor impairment, helping patients achieve their maximum level of functional autonomy and fulfill desired and required life roles after stroke [10]. Although an early OT intervention after stroke and discharge planning is considered an evidence-based intervention in many European countries [11–13], replication studies are needed to provide an evidence base in other countries and cultures. Thus, we examined the effectiveness of early OT intervention in a cohort of adults who suffered a stroke in a region of Spain with an extensive protocol evaluation with the final intention of introducing a novel intervention for the mentioned sanitary context.

Consequently, the primary objective of the present trial was to evaluate the effect of an Early Occupational Therapy Intervention Post-Stroke (EOTIPS) in the process of hospital discharge post-stroke on the patient quality of life within the Spanish public healthcare system, compared to a control group that received conventional rehabilitation and care. Secondary objectives were to assess improvements in functional independence, sensory–motor skills, perceptual–cognitive skills, communication skills and levels of depression and anxiety.

## Materials and methods

### Study design

EOTIPS was a prospective, longitudinal, randomized and controlled clinical trial, which protocol was previously defined and approved by the Malaga Research Ethics Committee (CEI) on 25^th^ February 2021 (see S1 and S2 Files). Patients’ evaluations within the EOTIPS research took place between May 2021 to November 2022. This study was registered on ClinicalTrials.gov with the identifier NCT04835363 and fully anonymous dataset was published [14]. Results are reported in accordance to CONSORT guidelines (see S3 File) [15].

### Participants and recruitment procedures

Recruitment started the 25^th^ May 2021 and finished the 30^th^August 2022. Patients assigned to the experimental group were included in EOTIPS, a program in which they received early occupational therapy intervention, and compared with a control group. Both groups received the usual care and rehabilitation provided by other health professionals within the public healthcare system. The rehabilitation and neurology departments were responsible for selecting and referring suitable patients to the occupational therapist to participate in the research. Only those who signed a written informed consent form were included in this study.

Participants were individuals that suffered a stroke and were admitted to the neurology ward of Virgen de la Victoria University Hospital (Malaga, Spain). The inclusion criteria were as follows: (1) diagnostic confirmation of stroke with single or multiple vascular lesions that have occurred in the same time period, demonstrated by neuroimaging tests (CT or MRI); (2) age > 18 years old; (3) >2 to <26 points on the National Institute of Health (NIHSS) scale; (4) 30–100 points on the Barthel Index (BI) on the second day after the stroke (with BI 100, the patient could be included if the Montreal Cognitive Assessment was<26); (5) going home on discharge, hence not going to a nursing home or rehabilitation unit. Inclusion in the study occurred prior to hospital discharge, and EOTIPS intervention started as soon as patients were medically fit for it and initial assessment was completed, in all cases within two weeks after the stroke. Therefore, this research took place exclusively with early sub-acute post-stroke patients.

Exclusion criteria were: (1) NIHSS> 26; (2) life expectancy <1 year; (3) having suffered a previous stroke, dementia or other types of illnesses associated with major neurocognitive disorders and other concomitants neurological, psychiatric or medical illnesses (for example, severe epilepsy, head trauma, schizophrenia, chronic obstructive pulmonary disease, severe or unstable heart disease or sleep apnea) that could alter cognitive function; (4) presence of moderate-severe aphasia and (5) people who do not understand Spanish or English.

Masked medical staff assessed participants inclusion and exclusion criteria and referred them to the evaluators for inclusion in the study. Eligible patients were randomly assigned to EOTIPS or the control group according to a computer pre-stablished designation in blocks of six patients, developed by the principal investigator of the study with the online free tool Sealed Envelope, v1.23.0 [16]. Regarding to the blinding process, evaluators and participants were masked to group allocation during the whole process. To minimize bias, participants were explained that they would enter an occupational therapy related study within which they would receive specialized monitoring of their stroke evolution. Only the occupational therapist leading the interventions was unblinded and explained every participant the intervention they were being provided, not revealing their group allocation. The statistical analysis was carried out by a blinded biostatistics expert and supervised by the principal investigator.

Sociodemographic and clinical variables were taken into account: age, sex, education, marital status, nationality, native language, employment status, stroke type, vascular territory of the lesion and Oxfordshire classification (see Table 1). Neurological impairments related to stroke were also contemplated and classified according to a recent classification system that divides the body impairments in four types: consciousness, cognition (includes disorientation, aphasia and inattention), motor (includes facial palsy, ataxia, dysarthria and dysphagia) and sensory impairments (includes eye movement and visual field impairments) [17]. This classification defines impairments using the National Institute of Health Stroke Scale (NIHSS) and their identification was done through the neurologist usual clinical practice examination who evaluates the patient systematically (see Table 3).

**Table 1:** Sociodemographic characteristics and clinical patients’ data at baseline.

	Control (N = 30)	Experimental (N = 30)	Total (N = 60)	p value
**SEX**				0.432^1^
Female	14.00 (46.66%)	11.00 (36.66%)	25.00 (41.66%)	
Male	16.00 (53.33%)	19.00 (63.33%)	35.00 (58.33%)	
**AGE**				0.479^2^
Mean (SD)	66.60 (10.60)	68.50 (10.70)	67.50 (10.60)	
**EDUCATION**				0.299^1^
No studies	1.00 (3.33%)	1.00 (3.33%)	2.00 (3.33%)	
Knows how to read and write	7.00 (23.33%)	11.00 (36.66%)	18.00 (30.00%)	
Primary School	15.00 (50.00%)	9.00 (30.00%)	24.00 (40.00%)	
High School	5.00 (16.66%)	3.00 (10.00%)	8.00 (13.33%)	
University	2.00 (6.66%)	6.00 (20.00%)	8.00 (13.33%)	
**MARITAL STATUS**				0.974^1^
Single	2.00 (6.66%)	3.00 (10.00%)	5.00 (8.33%)	
Divorced	2.00 (6.66%)	2.00 (6.66%)	4.00 (6.66%)	
Widowed	5.00 (16.66%)	5.00 (16.66%)	10.00 (16.66%)	
Married/living with partner	21.00 (70.00%)	20.00 (66.66%)	41.00 (68.33%)	
**NACIONALITY**				0.278^1^
Spain	27.00 (90.00%)	24.00 (80.00%)	51.00 (85.00%)	
Other countries	3.00 (10.00%)	6.00 (20.00%)	9.00 (15.00%)	
**NATIVE LANGUAGE**				1.000^1^
Spanish	28.00 (93.33%)	28.00 (93.33%)	56.00 (93.33%)	
Non-Spanish	2.00 (6.66%)	2.00 (6.66%)	4.00 (6.66%)	
**EMPLOYMENT STATUS**				0.507^1^
Househusband/Housewife	1.00 (3.33%)	4.00 (13.33%)	5.00 (8.33%)	
Unemployed	2.00 (6.66%)	1.00 (3.33%)	3.00 (5.00%)	
Retired	19.00 (63.33%)	17.00 (56.66%)	36.00 (60.00%)	
Temporary disability	8.00 (26.66%)	7.00 (23.33%)	15.00 (25.00%)	
Other activities	0.0 (0.00%)	1.00 (3.33%)	1.00 (1.66%)	
**STROKE TYPE**				0.052^3^
Ischemic	25.00 (83.33%)	30.00 (100.00%)	55.00 (91.66%)	
Hemorrhagic	5.00 (16.66%)	0.00 (0.00%)	5.00 (8.33%)	
**DAMAGED HEMISPHERE**				0.400^1^
Left	15.00 (50.00%)	19.00 (63.33%)	34.00 (56.66%)	
Right	14.00 (46.66%)	11.00 (36.66%)	25.00 (41.66%)	
Both hemispheres	1.00 (3.33%)	0.00 (0.00%)	1.00 (1.66%)	
**OXFORDSHIRE CLASSIFICATION**				0.893^1^
LACS	16.00 (53.33%)	15.00 (50.00%)	31.00 (51.66%)	
PACS	11.00 (36.66%)	10.00 (33.33%)	21.00 (35.00%)	
POCS	2.00 (6.66%)	3.00 (10.00%)	5.00 (8.33%)	
TACS	1.00 (3.33%)	2.00 (6.66%)	3.0 (5.00%)	
BASELINE EVALUATIONS*				
**NIHSS**	6.60 (5.30)	4.60 (3.00)	5.60 (4.44)	0.197^4^
**SAQOL-39**	2.51 (0.75)	2.67 (0.72)	2.59 (0.73)	0.102^4^
**Barthel Index**	52.50 (21.00)	54.70 (20.50)	53.60 (20.60)	0.343^2^
**Modified Rankin Scale**	3.60 (0.77)	3.63 (0.80)	3.62 (0.78)	0.445^4^
**MoCA**	16.60 (7.33)	18.60 (7.16)	17.60 (7.25)	0.149^2^
**BBS**	22.70 (17.90)	27.60 (14.60)	25.20 (16.40)	0.229^4^
TUG*	28.40 (17.70)	29.00 (12.90)	28.70 (14.70)	0.709^4^
**FMA (upperlimbsection)**	99.80 (22.60)	102.00 (18.90)	101.00 (20.70)	0.521^4^
**SIS-16**	36.60 (14.00)	37.70 (11.00)	37.10 (12.50)	0.132^4^
**CAL**	109.00 (37.00)	122.00 (27.30)	115.00 (32.90)	0.100^4^
**BDI-II**	13.6 (9.76)	11.2 (9.11)	12.40 (9.44)	0.193^4^
**HAM-A**	11.50 (10.90)	8.47 (11.00)	10.00 (10.90)	0.059^4^

1. Pearson’s Chi-squared test.

2. Student’s T-test.

3. Fisher’s exact test.

4. Non-parametric Mann–Whitney *U* test.

BASELINE EVALUATIONS*: Datapresented as Mean (SD).

TUG*: Only participants that completed both initial and final evaluation have been included in the statistical analysis (n = 34).

**Abbreviations:**SAQOL-39 = Spanish Stroke and Aphasia Quality of Life Scale; MoCA = Montreal Cognitive Assessment; BBS = Berg Balance Scale; TUG = Timed Up and Go; FMA = Fugl-Meyer Assessment; SIS-16 = Stroke Impact Scale-16; CAL = Communicative Activity Log; BDI-II = Beck Depression Inventoryand Aphasia Quality of Life Scale; HAM-A = Hamilton anxiety scale; LACS = Lacunar Syndrome; PACS = Partial anterior Circulation Stroke; POCS = Posterior Circulation Stroke; TACS = Total Anterior Circulation Stroke.

During data collection, the anonymity of users was guaranteed. Participants were informed of the intervention and were asked to sign a written informed consent, since the participation was voluntary, allowing the user to leave whenever they wished. The principles of the Declaration of Helsinki were followed [18].

### Interventions

EOTIPS main objective was to improve patients’ quality of life facilitating the transition from hospital to home. The intervention group received EOTIPS delivered by a single expert occupational therapist, who had advanced training in neurology. It took place both in the hospital and the patient’s home. Before beginning rehabilitation, the occupational therapist and their family explored the needs and wishes of the patient and set individual goals for the intervention period [19].

**Fig 1:**
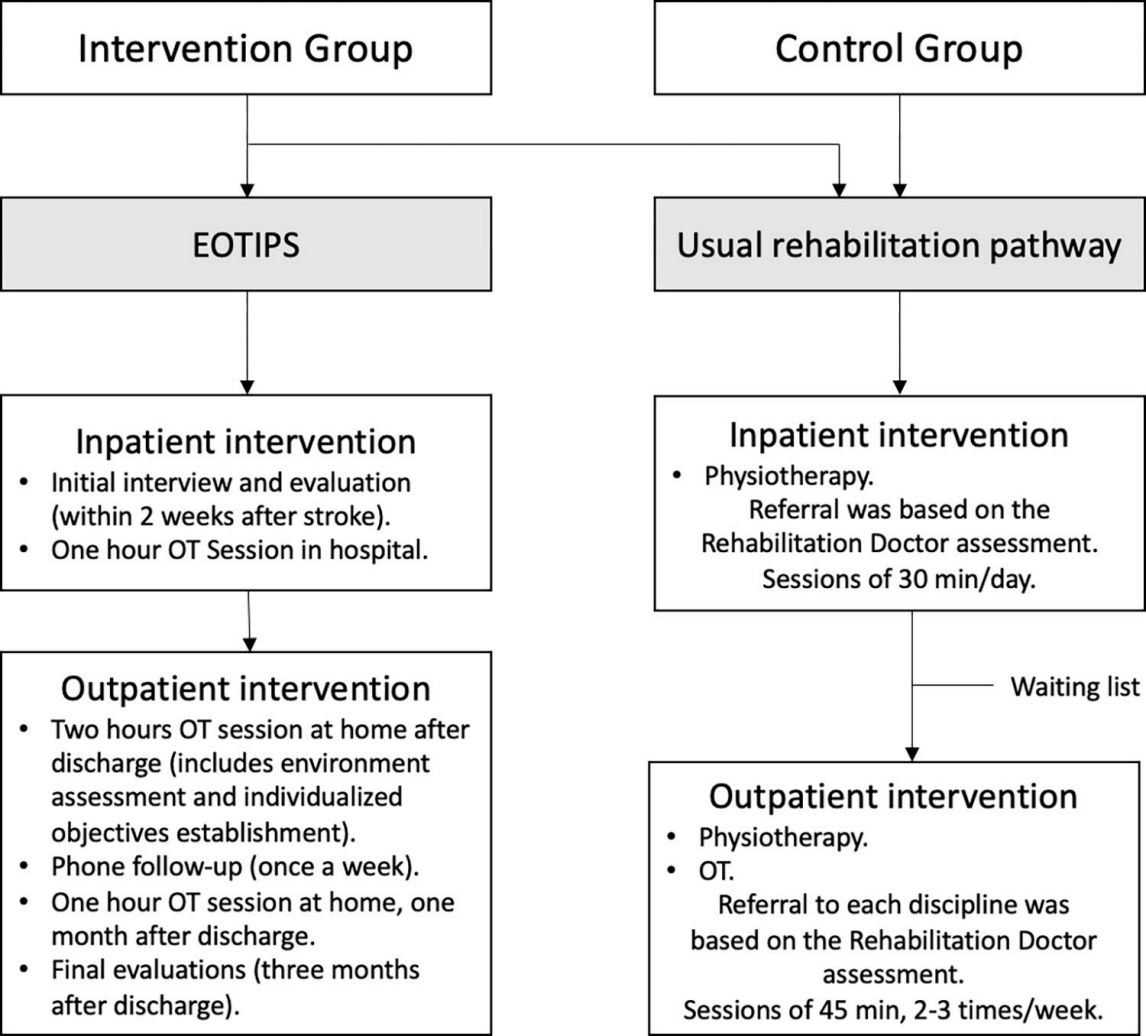
Rehabilitation pathway.

The intervention had a person-centered approach, which is inherent to occupational therapy, that was based on the patient’s context and history, their individual strengths and weaknesses and social support network. Therefore, individualized objectives were established, taking into account their abilities and limitations. EOTIPS included an initial evaluation, a first session in hospital, a post-discharge home visit, a home visit one month later, and a final evaluation three months after discharge. Although all patients received advice to continue rehabilitation at home following the occupational therapist guidance, frequency and intensity of practice they performed at home depended on their willingness to improve and adherence to treatment. The occupational therapistmaintained communication with patients and caregivers via home visits and phone calls to offer information and support during the transition from hospital to home. The overall aim was to educate patients with information and knowledge about the importance of postural care, cognitive/motor/perceptual/sensitive stimulation and basic neuro-rehabilitation to promote involvement in the intervention and, consequently, enhancing the participation and engagement in therapeutic activities. This intervention was carried out in parallel to the standard of care provided by the public healthcare system when the trial took place (see Fig 1). During the study, patients were evaluated by the rehabilitator doctor and referred to physiotherapy or OT depending on their needs and abilities. Nevertheless, in the hospital where the intervention and during the time the study took place, OT was not provided in the ward or at the patient´ home, being only provided in the OT department for outpatients who were previously referred by the rehabilitator doctor.

### Outcomes

After determining potential participants’ eligibility, evaluators facilitated a research information document and proceeded with initial evaluations. Evaluators were OT practitioners previously trained to carry out the assessments and who were blinded to group assignment. Initial assessments were performed in hospital while final assessments took place at patients’ homes. All measures used in the research were standardized and validated for stroke population and adapted to Spanish language [20–39].

Clinical and social data were obtained for all participants at each evaluation:

The primary outcome was quality of life, assessed by the Stroke and Aphasia Quality of Life Scale (SAQOL-39) [20–22]. In this scale, the 39 items are scored from one to five (higher score means better performance). Items scores are added and divided by 39, obtaining a result between zero and five where higher final score indicates better quality of life.

Level of functional independence was measured by Barthel Index (BI), Modified Rankin Scale (mRS) and Stroke Impact Scale-16 (SIS-16). BI measures the extent to which someone can function independently during basic ADL. Each performance item is rated on this scale with a given number of points assigned to each level. The modified version with 0–100 was used in this study, where a lower score indicates higher dependence [23]. mRS is used to describe disability in general. The scale ranges from 0 (perfect health) to 6 (death) [24]. SIS-16 is a reduced version of the Stroke Impact Scale version 3.0. Items are scored on 5-grade ordinal scale, 1 meaning the activity could not be completed and 5 meaning it was completed with no difficulties. The scale ranges from 16 (dependent) to 80 (independent) [25–27].

Sensory-motor skills were assessed by Fugl Meyer Assessment (FMA), Berg Balance Scale (BBS) and Timed Up & Go (TUG). We used the Spanish version of the FMA to evaluate the motor and sensory progress of the affected upper limb, therefore, we did not include the lower limb section of the scale (maximum score of 126). FMA is a scale created to measure motor recovery after stroke. Items are scored on a 3-grade ordinal scale, with 0 as the minimum and 2 as the maximum [28–30]. BBS assesses functional balance. Performance on this test is rated from 0 (cannot perform) to 4 (normal performance) on 14 different tasks. The maximum score is 56 and higher score indicates better balance skills [31]. TUG assesses basic mobility, counting the time required for a person to get up from a standardized chair, walk a distance of three meters, turn, return to the chair, and sit down again. A shorter time indicates better performance [32].

Perceptual-cognitive skills were assessed by Montreal Cognitive Assessment (MoCA). MoCA is a brief screening instrument with good predictive value for the development of cognitive impairment after stroke. The assessment scores from 0 to 30, being 30 the highest score that means absence of cognitive impairment [33–36].

Communication skills were assessed by Communicative Activity Log (CAL). CAL scale allows obtaining information on communication skills in activities of daily life referring to comprehensive and expressive aspects of language. It is made up of 36 items that assess both the quality and the quantity of the patient’s communication. The performance is obtained through the sum of the scores in each item [37].

Mood disorders (levels of anxiety and depression), were assessed by Beck Depression Inventory–II (BDI-II)and Hamilton Anxiety Scale (HAM-A). BDI-II is a self-administered questionnaire that consists of 21 multiple-choice questions. It is one of the most commonly used instruments to measure the severity of depression. The maximum score is 63 (higher score, higher level of depression) [38]. HAM-A assesses the severity of anxiety globally in patients who meet criteria for anxiety or depression. It is made up of 14 items, 13 of which refer to anxious signs and symptoms and the last one that assesses the patient’s behavior during the interview [39].

### Statistical analysis

The sample size ″n″ was intended to represent the total population of patients who suffer a stroke admitted to a second level hospital in Málaga (Spain) and who maintain sequelae susceptible to rehabilitation. It was calculated based on the primary outcome SAQOL-39 [20–22] with the sample size determination formula for two independent groups, assuming a mean of 2.50, standard deviation of 0.75, significance level of 5%, a power of 80% and a critical difference of 0.54, obtaining a sample of 30 participants per group, and a total of 60 participants. Therefore, two groups were formed: an intervention group of 30 patients who received the Early Occupational Therapy program (EOTIPS) in addition to conventional rehabilitation and care, and a control group of 30 patients who received conventional rehabilitation and care.

The power was finally recalculated with the G*Power tool [40] assuming SAQOL-39 as the primary outcome, an effect size of 0.3, a sample size of 30, a calculated measurement coefficient of 0.654, two repeated measures and two groups, obtaining a final power in the study of 96%.

Sociodemographic and clinical variables were reported using the mean and standard deviation in case of quantitative variables; and number and percentage for qualitative variables. Baseline differences between the two treatment groups were assessed by Student’s T-test or non-parametric Mann-Whitney U test according to normality deviation tests when variables were quantitative, and Pearson’s Chi-squared test or Fisher’s exact test as appropriate when variables were qualitative.

In order to compare changes between the control and the experimental group a repeated measures ANOVA was performed considering one within-factor (Time) and one between-factor (Group). Where ANOVA factors were significant, post-hoc pairwise comparisons were performed to identify comparisons driving the differences. As the variable “Type of stroke” was almost significant, it was considered as a possible confusion factor. For all tests, a *P* value of < .05 was considered statistically significant. For the statistical analysis of ordinal variables, non-parametric tests were used. For the other variables, the conditions of normality and homoscedasticity (equality of variances) were contrasted. The software used for data analysis was JAMOVI, version 2.3.26 [41].

## Results

### Recruitment and participant characteristics

**Fig 2:**
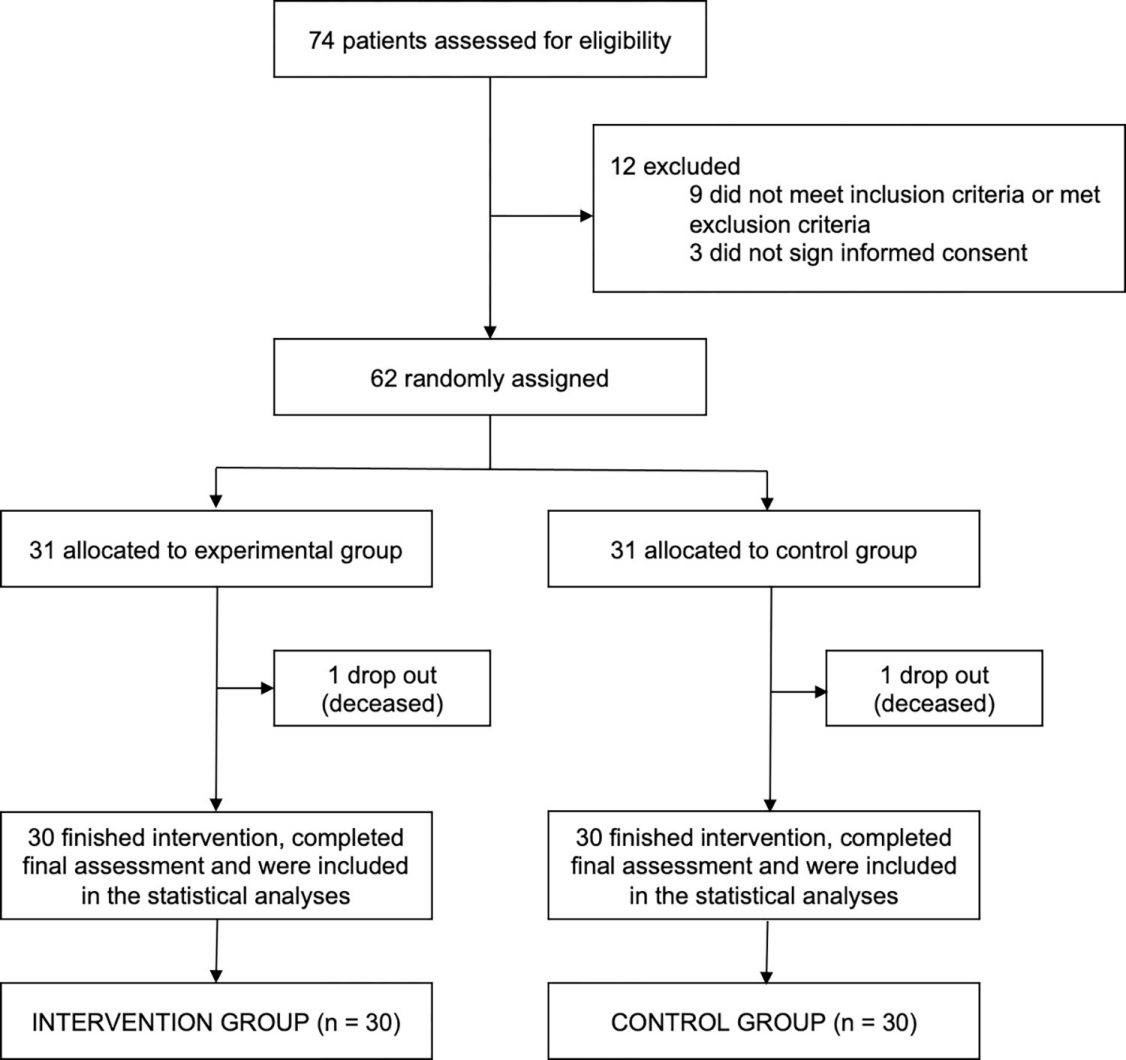
Trial profile.

We screened 74 potential participants, of whom 65 were eligible to participate in the trial; three patients declined to sign the written informed consent. Therefore, 62 participants were randomly assigned to the intervention and control groups (31 per group): 1 intervention participant and 1 control participant deceased, not concluding the study. An overview of the flow of participants through the study is provided in Fig 2.

Recruitment ended after 15 months, once the sample size was accomplished. The final sample thus included 60 participants. The intervention and control group did not present significant differences on the sociodemographic characteristics and clinical data at baseline, being the average age 67.5 (±SD 10.60) and male/female ratio of 1.4 (see Table 1). Length of hospitalization varied between 3 and 30 days, being the mean 10 days and standard deviation 6.31. Therefore, all participants were in the early sub-acute phase of their first stroke (see Table 2).

**Table 2:** Time from hospital admission to initial evaluation.

	Control (N = 30)	Experimental (N = 30)	Total (N = 60)	p value
Mean (SD)	4.40 (3.10)	5.20 (3.10)	4.80 (3.10)	0.267^1^
Range	1.00–13.00	1.00–14.00	1.00–14.00	

1. Non-parametric Mann–Whitney *U* test.

**Time from hospital admission to initial evaluation (counted in days):** Data presented as Mean (SD).

In regards to race, 100% of the study were Caucasian. Although the major ethnicity that entered the study was Spanish (93.3%), other ethnicities entered in the sample, such as Latin-American (6.66%), Portuguese (1.7%), Arabs (3.33%) and White British (3.33%).

The most common type of stroke within the participants was ischemic in opposition to hemorrhagic (χ^2^ = 5.45, *p* = 0.052), not showing statistically significant difference between damaged hemispheres (χ^2^ = 1.83, *p* = 0.400). There were not significant differences between groups in any of the baseline evaluations.

Regarding the participants neurological impairments related to stroke, no significant differences were found for any of the categories: consciousness (p = 1.000), cognitive (p = 0.771), motor (p = 1.000) and sensory impairments (p = 0.792)(see Table 3).

**Table 3:** Participants neurological impairments.

	Control group (n = 30)	Experimental group (n = 30)	P value
**Consciousness**	2 (3.33%)	2 (3.33%)	1.000^1^
**Cognitive impairments**	7 (11.66%)	9 (15.00%)	0.771^1^
**Motor impairments**	29 (48.33%)	30 (50.00%)	1.000^1^
**Sensory impairments**	11 (18.33%)	17 (28.33%)	0.792^1^

1. Fisher’s exact test.

Data presented as Count (% of the total participants).

### Summary of participants individual goals

Before the start of EOTIPS intervention, the occupational therapist explored the needs and wishes of the patient and together decided on individual goals for the intervention period (examples of chosen goals, both general and specific, are shown in Table 4).Most of the chosen general objectives were related to basic and instrumental ADL. However, they were also related to social participation and significant activities which is, in turn, their sense of self and occupational identity [42, 43].

**Table 4:** General and specific objectives proposed by experimental group participants.

*General objectives*	Total of patients that decided on this general objective	Number of patients that met the goal at the end of treatment
*Getting dressed independently*	7	6
*Preparing small meals independently*	9	7
*Having a shower/bathing independently*	6	5
*Being able to eat independently*	3	3
*Being able to cut with knife and fork*	1	1
*To improve computer typing*	1	1
*Going to the bakery and buying bread independently*	1	1
*Shopping*	2	2
*Going back to work*	4	2
*Functional mobility at home*	4	4
*Functional mobility outdoors*	7	6
*To improve transfers (chair-to-bed; bed-to-chair; chair-to-chair)*	3	3
*To improve communication skills*	3	3
*Being able to stay alone at home*	3	3
*Toileting independently*	3	3
*Home duties*	2	2
*Walking up/down the stairs*	3	3
*To drive again*	3	2
*Sleeping routine*	1	0
*Looking after parents*	1	1
*To participate in significant activity*	1 (photography)	0
	1 (reading a book)	1
	3 (gardening)	2
	1 (going to pensioners club)	1
	2 (going for a walk)	1
***Specific objectives***	**Total of patients that decided on this specific objective**	**Number of patients that met the goal at the end of treatment**
*To improve exercise tolerance*	15	13
*To improve balance and safety when walking*	14	12
*To improve affected UL sensibility*	1	1
*To improve affected LL sensibility*	1	0
*To improve sensitivity of the affected hemibody*	1	0
*To improve strength and motor control of affected UL*	9	9
*To improve strength and motor control of affected LL*	4	4
*To improve attention on affected hemibody*	3	3
*To improve trunk control while sitting*	1	1
*To improve attention*	2	2
*To improve memory*	1	1
*To improve visuo-special skills*	2	2
*To increase wrist range of motion*	1	1
*To improve manual dexterity*	10	9
*To improve dysarthria / motor aphasia*	2	2

*LL: Lower Limb; UL: Upper Limb.

## Outcome analysis

**Fig 3:**
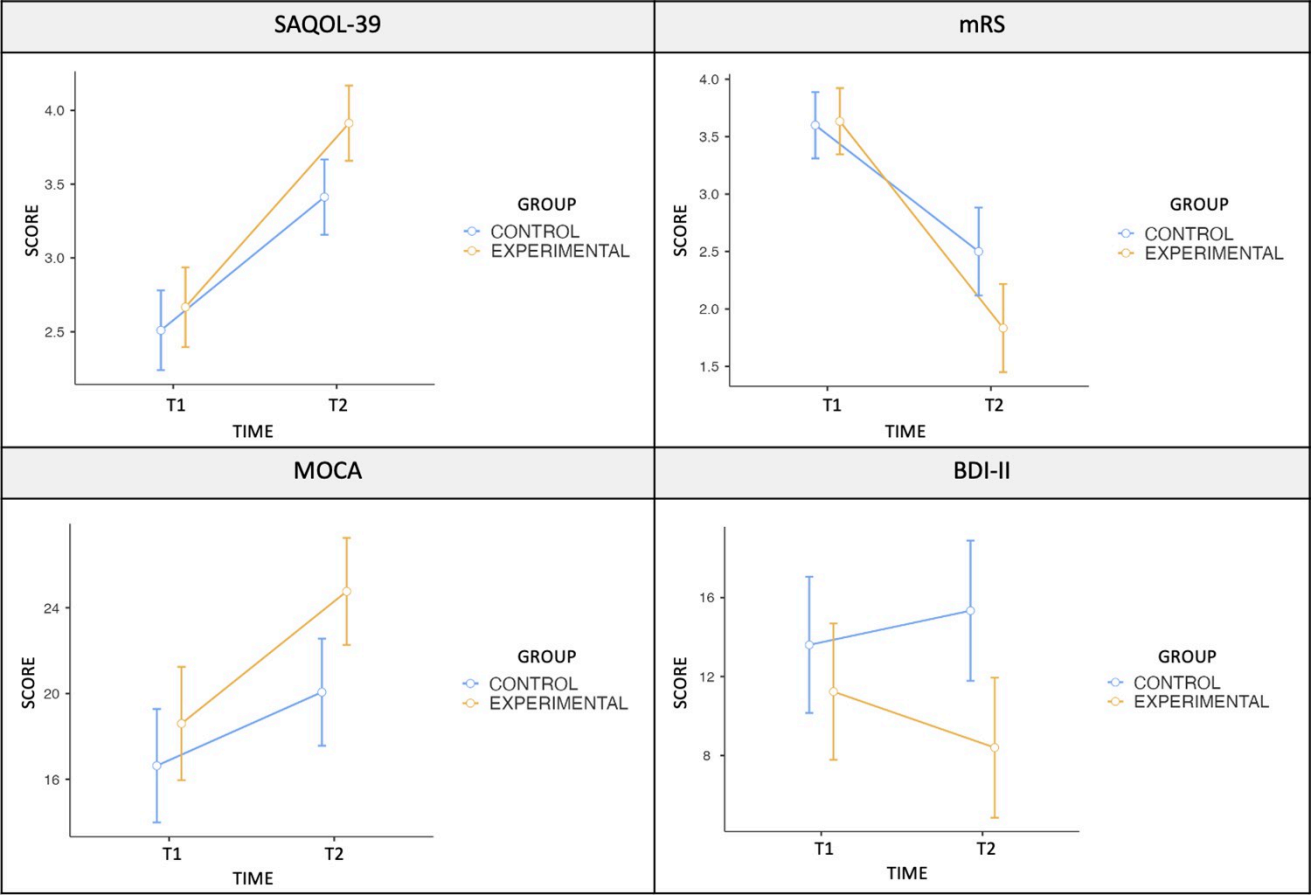
Significant outcome measures.

Table 5 shows the results of a repeated measures ANOVA which was conducted to examine the change of the variables from baseline to final evaluation on the evaluations scores. In 4 of the variables analyzed there was a significant interaction between the time of evaluation (within-subject factor) and group (between-subjects factor), therefore their results of the Tukey post-hoc Test have been added in the last column, revealing statistically significant differences for three of them. The means and standard deviations in the initial and final evaluations for each group have been included, as well as the analysis results to verify significant differences between both groups, including p values, F values and degrees of freedom (gl). The mean percentage of change (%) has also been indicated to better understand the magnitude of the improvement or worsening in relation to the baseline evaluation (see Table 5). In addition, Fig 3 shows the evolution of those variables in which the interaction factor (Time*Group) has been significant (see Fig 3).

**Table 5:** Results of repeated measures ANOVA for the outcome measures.

Repeated Measures ANOVA								
		Control Group	Experimental Group	Time	Group	Treatment*Time	POST-HOC	
				Within-subjects factor	Between-subjects factor	Interaction	Exp vs Cont	
		Mean (SD)	Mean (SD)					
**SAQOL-39**	**T1**	2.51 (0.75)	2.67 (0.72)	F = 197.19	F = 3.78	F = 5.02	T1	0.843^3^
	**T2**	3.41 (0. 69)	3.91 (0.70)	gl = 1.58	gl = 1.58	gl = 1.58	T2	**0.036** ^3^
	**Change (%)**	18.00%	24.00%	p = < .001^1^	p = 0.057^1^	p = **0.029**^**1**^		
**Barthel Index**	**T1**	52.50 (21.00)	54.70 (20.50)	F = 268.95	F = 0.69	F = 0.46		
	**T2**	85.00 (16.70)	90.00 (14.90)	gl = 1.58	gl = 1.58	gl = 1.58		
	**Change (%)**	32.50%	35.30%	p = < .001^1^	p = 0.407^1^	p = 0.496^**1**^		
**mRS**	**T1**	3.60 (0.77)	3.63 (0.80)	F = 154.04	F = 0.13	F = 8.97	T1	0.998^3^
	**T2**	2.50 (1.04)	1.83 (1.05)	gl = 1.58	gl = 1.58	gl = 1.58	T2	0.076^3^
	**Change (%)**	15.71%	25.71%	p = < .001^1^	p = 0.135^1^	p = **0.004**^**1**^		
**MoCA**	**T1**	16.6 (7.33)	18.6 (7.16)	F = 83.91	F = 3.67	F = 6.80	T1	0.720^3^
	**T2**	20.1 (7.06)	24.8 (6.58)	gl = 1.58	gl = 1.58	gl = 1.58	T2	**0.047** ^3^
	**Change (%)**	11.66%	20.66%	p = < .001^1^	p = 0.060^1^	p = **0.012**^**1**^		
**BBS**	**T1**	22.70 (17.90)	27.60 (14.60)	F = 141.83	F = 1.08	F = 0.85		
	**T2**	45.30 (11.70)	47.00 (11.10)	gl = 1.58	gl = 1.58	gl = 1.58		
	**Change (%)**	40.35%	34.64%	p = < .001^1^	p = 0.304^1^	p = 0.358^**1**^		
TUG*	**T1**	28.40 (17.68)	29.00 (12.90)	F = 93.27	F = 2.46	F = 4.94		
	**T2**	12.40 (4.83)	14.30 (12.80)	gl = 1.58	gl = 1.58	gl = 1.58		
	**Change (%)**	58.20%	52.00%	p = < .001^1^	p = 0.747^1^	p = 0.797^**1**^		
**FMA (upperlimbsection)**	**T1**	99.80 (22.60)	102.00 (18.90)	F = 75.97	F = 0.82	F = 2.15		
	**T2**	111.10 (17.60)	117.00 (12.60)	gl = 1.58	gl = 1.58	gl = 1.58		
	**Change (%)**	8.88%	11.90%	p = < .001^1^	p = 0.367^1^	p = 0.148^**1**^		
**SIS-16**	**T1**	36.60 (14.00)	37.70 (11.00)	F = 367.60	F = 0.95	F = 1.40		
	**T2**	61.90 (12.60)	66.30 (10.70)	gl = 1.58	gl = 1.58	gl = 1.58		
	**Change (%)**	31.60%	35.75%	p = < .001^1^	p = 0.333^1^	p = 0.241^**1**^		
**CAL**	**T1**	109.00 (37.00)	122.00 (27.30)	F = 30.17	F = 5.04	F = 3.23		
	**T2**	116.00 (31.10)	137.00 (24.20)	gl = 1.58	gl = 1.58	gl = 1.58		
	**Change (%)**	3.88%	8.33%	p = < .001^1^	p = 0.029^1^	p = 0.078^**1**^		
**BDI-II**	**T1**	13.60 (9.76)	11.23 (9.11)	F = 0.40	F = 4.03	F = 6.93	T1	0.767^3^
	**T2**	15.30 (10.48)	8.40 (8.88)	gl = 1.58	gl = 1.58	gl = 1.58	T2	**0.037** ^3^
	**Change (%)**	2.69%	-4.44%	p = 0.528^1^	p = 0.049^1^	p = **0.011**^**1**^		
**HAM-A**	**T1**	11.50 (10.88)	8.47 (10.96)	F = 3.79	F = 3.38	F = 3.79		
	**T2**	11.30 (9.40)	5.50 (7.94)	gl = 1.58	gl = 1.58	gl = 1.58		
	**Change (%)**	-0.35%	5.30%	p = 0.528^1^	p = 0.809^2^	p = 0.055^2^		

1. Repeated measures ANOVA.

2. Repeated measures ANOVA including confusion factor of Oxfordshire Classification variable in the analysis.

3. Tukey test.

**Abbreviations:**SAQOL-39 = Spanish Stroke and Aphasia Quality of Life Scale; mRS = modified Rankin Scale; MoCA = Montreal Cognitive Assessment; BBS = Berg Balance Scale; TUG = Timed Up and Go; FMA = Fugl-Meyer Assessment; SIS-16 = Stroke Impact Scale-16; CAL = Communicative Activity Log; BDI-II = Beck Depression Inventory; HAM-A = Hamilton anxiety scale.

TUG*: Only participants that completed both initial and final evaluation have been included in the statistical analysis (n = 34).Percentage of change was calculated taking into account the longest time score.

**Fig 4:**
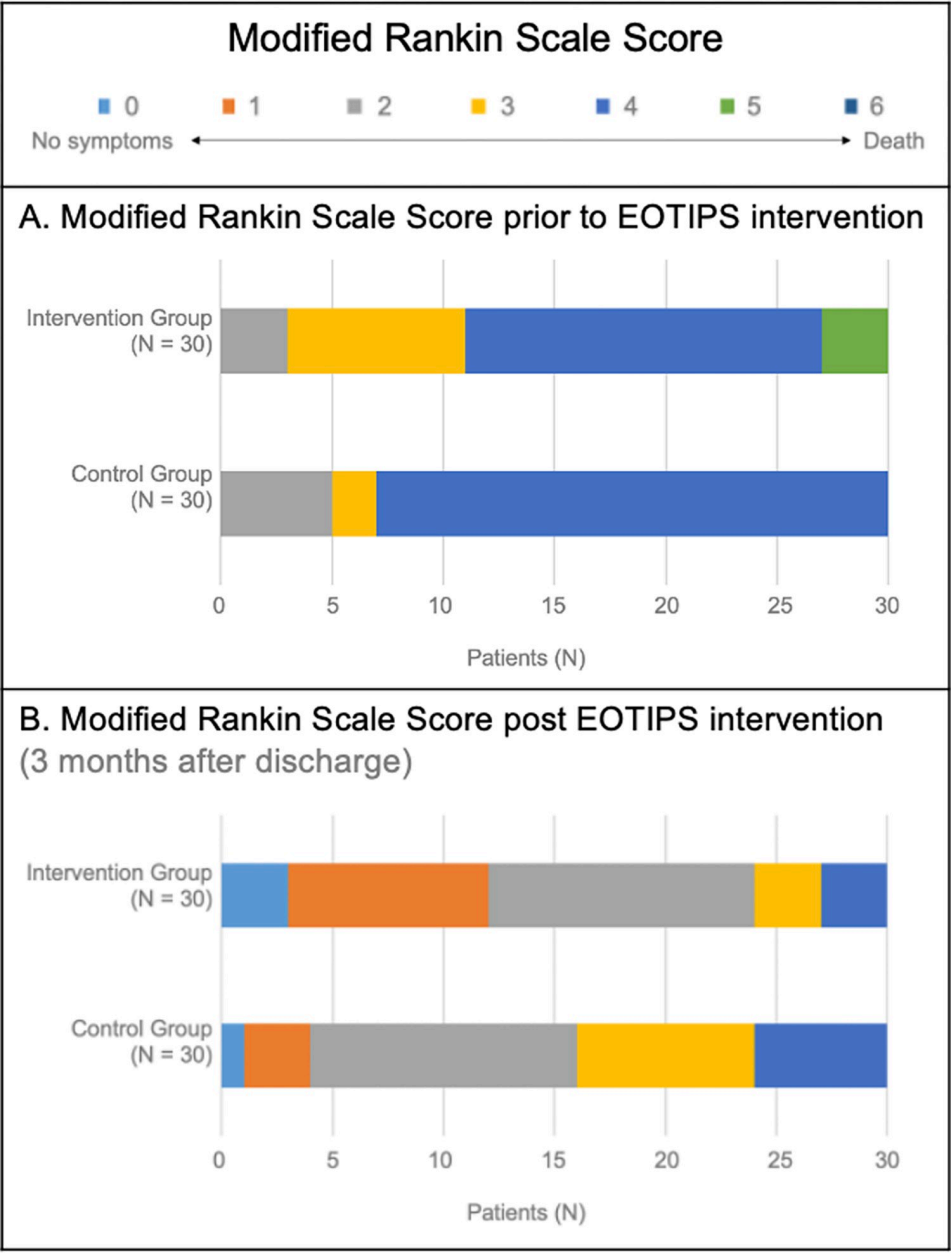
mRS graphic comparing pre-post score results.

Repeated measures ANOVA results indicated that mean change in the pre-post evaluation for four of the evaluation scores were significantly different between groups, being those evaluations SAQOL-39 (*F* = 5.02, *p* = 0.029), mRS (F = 8.97, p = 0.004), MOCA (F = 6.80, p = 0.012) and BDI-II (F = 6.93, p = 0.011). Post-hoc pairwise comparisons revealed that evaluations scores were not significantly different between the control and the experimental group for the baseline evaluation (T1), but were for the final evaluation (T2)ofthe scalesSAQOL-39 (T1, p = 0.843; T2, *p* = 0.036), MOCA (T1, p = 0.720; T2, *p =* 0.047) and BDI-II (T1, p = 0.767; T2, *p* = 0.037); but was not for mRS (T1, p = 0.998; T2, *p =* 0.076). Modified Rankin Scale (mRS) scores significantly improved for both groups (p = < .001), as shown by the statistical analysis of Wilcoxon signed-rank test (see Table 6). Therefore, evaluations related to functional independence, quality of life, cognition skills and level of depression showed better recovery for the experimental group (see Figs 3 and 4).

**Table 6:** Modified Rankin scale analysis.

		0	1	2	3	4	5	6	Total	Change T1-T2 (%)	P value
**Control group**	T1	0 (0.00%)	0 (0.00%)	5 (16.66%)	2 (6.66%)	23 (76.66%)	0 (0.00%)	0 (0.00%)	30 (100.00%)	15.71%	**< .001** ^1^
	T2	1 (3.33%)	3 (10.00%)	12 (40.00%)	8 (26.66%)	6 (20.00%)	0 (0.00%)	0 (0.00%)	30 (100.00%)		
**Experimental group**	T1	0 (0.00%)	0 (0.00%)	3 (10.00%)	8 (26.66%)	16 (53.33%)	3 (10.00%)	0 (0.00%)	30 (100.00%)	25.71%	**< .001** ^1^
	T2	2 (6.66%)	10 (33.33%)	12 (40.00%)	3 (10.00%)	3 (10.00%)	0 (0.00%)	0 (0.00%)	30 (100.00%)		

1. Non-parametric Wilcolxon.

Data presented as Count (%).

**Abbreviations:** T1 (Time 1, meaning initial evaluation); T2 (Time 2, meaning final evaluation).

**mRS description:** 0—No symptoms; 1 –No significant disability despite some symptoms (able to carry out all duties and activities) 2—Slight disability (Unable to carry out all previous activities, but able to look after own affairs without assistance); 3—Moderate disability (requiring some help, but able to walk without assistance); 4 –Moderately severe disability (unable to walk without assistance and unable to attend own bodily needs without assistance.

It is noteworthy that both groups, having participated or not in the EOTIPS program in addition to conventional rehabilitation, greatly improved scores on all evaluations comparing T1 and T2 scores. Mean change scores for the secondary scaled goals were greater for the intervention group compared with the control group in most variables, although not achieving significant differences (see Table 5): independence, as scored by Barthel Index (F = 0.46, p = 0.496); general independence after stroke, as scored by SIS-16 (F = 1.40, p = 0.241); upper limb function, as scored by FMA (F = 2.15, p = 0.148); level of anxiety, as scored by HAM-A (F = 3.79, p = 0.055) and communication skills, as scored by CAL (F = 3.23, p = 0.078).Nevertheless, mean change scores for functional balance, as shown BBS, were higher for the control group than the experimental group (F = 0.85, p = 0.358). Due to the inability of some participants to stand up during the initial evaluation (43.3%), TUG test could not be completed. Therefore, only participants that completed both initial and final evaluation could be included in the statistical analysis (n = 34). However, it must be highlighted that 21 patients of the experimental group against 13 of the control group succeeded in completing the TUG test. Finally, it is worth to mention no harms or unintended negative effects were generated or reported during the trial.

## Discussion

This study presents the development of a RCT based on an early OT intervention during the hospital discharge post-stroke. To our knowledge, it is the first prospective, randomized clinical trial to investigate the effect of adding an early OT discharge planning intervention that involves home visits and goal setting in the Spanish public healthcare system. The trial had random allocation, blinded assessments and intention-to-treat analysis. This research has relevance as it provides evidence supporting an intervention that addresses challenges found at home after discharge and emphasizes the importance of a personal-centered approach [44–46].

Studies with an evaluation protocol as extensive and complete are not common [47]. Likewise, no similar studies have been found assessing quality of life as the main efficacy variable. However, there are articles that have included it as a secondary variable and have shown significant improvements after the intervention [4]. Furthermore, this research exposed cohesion with other published articles significant results: improvements in functional independence [48–50], cognition skills [51] and symptoms of depression [5]. Most participants met their chosen goals at the end of EOTIPS due to regained ability for doing the activity and/or to activities/tools adaptation to perform the task safely, which resulted in higher participants satisfaction and quality of life. Nevertheless, according to other studies the effectiveness of the potential benefits of OT interventions remains unclear [52], being the need of further research the main motivation for the current study.

There are not many studies on the effectivity of a multidisciplinary intervention in the process of hospital discharge after stroke that includes the figure of the occupational therapist, with the majority being carried out in northern Europe [3, 4, 53–55], UK [56], China [12, 57] and Australia [47]. Some studies include the occupational therapist as part of the multidisciplinary services facilitated by an Early Supported Discharge (ESD) or a Very Early Supported Discharge (VESD) model, which has been proven to reduce long-term dependency and admission to institutional care [3, 56]. Although most of related researches incorporate caregivers as a potentiality in the patient rehabilitation [6], only 68.3% of participants were married or living with their partner who accepted to be deeply involved in their care.

In the current study, inclusion and exclusion criteria used were those usually described in previous researches but with an improved evaluation protocol that included valid and reliable clinical tools [47]. Decision on evaluations and intervention duration was made after reviewing recent and similar published papers, as showing main changes between groups 3 months after discharge [3, 4, 49, 53, 58] and being Barthel Index and the mRS the most widely used evaluations [3, 4, 53, 59]. Cognitive skills were also assessed as more than 40% of stroke survivors experience cognitive impairment (mild post-stroke cognitive impairment—MCI) soon after the event [57].

As expected, the participants had stroke severity at the mild end of the NIHSS scale, previously shown to be the target population of ESD intervention [56]. According to the Oxfordshire Community Stroke Project classification [60], the most common type of stroke within the RCT was lacunar syndrome (LACS), followed by partial anterior circulation stroke (PACS) and posterior circulation syndrome (POCS). However, only 3 patients with total anterior circulation stroke (TACS) were included in the study (being the 3% of the control group and the 7% of the experimental group). On the other hand, it seems important to highlight that there was a significant difference in stroke type, which may have influenced the final results as it has been generally believed that hemorrhagic strokes are generally more severe as within the first 3 months after stroke. Moreover, hemorrhagic strokes are associated with a considerable increase of mortality [61], although that did not show in our study, being mortality rate identical between groups. Nevertheless, despite hemorrhagic stroke patients usually presenting with worse functional and clinical status compared to ischemic stroke, there are studies that support the same or even better functional prognosis in stroke survivors with hemorrhagic stroke, with age and initial stroke severity as the main prognostic factors [62, 63]. Other studies consider that, although the pathophysiology of these types of strokes is different, both ultimately result in similar injuries, possibly accounting for lack of findings of functional levels differences between groups after rehabilitation [64].

The neurological impairments related to stroke were also contemplated and classified according to a new classification system [17], which shows most of them had motor impairments (98.33%) followed by sensory impairments (46.66%), cognitive impairments (26.66%) and consciousness related impairments (6.66%), not showing significant differences between groups. In general terms, there were no differences between groups at baseline. On the time of the study, in-patient rehabilitation consisted on nine beds and the decision was made by the rehabilitator doctor based on different reasons, such as severity of the stroke, rehabilitation potential, age, social support and bed availability. As this service is part of the public healthcare system, admission to rehabilitation unit happened at no economical cost. However, patients must have had relatives or friends to support them in the unit. Therefore, a strong support net was crucial for admission. Thus, people with severe stroke, strong social net and young age were more likely to be sent to rehabilitation as an inpatient. Nevertheless, patients going home on discharge must also have had social support or be independent enough to manage at home despite any limitation. Only 5 participants in total (2 in the experimental group and 3 in the control group) had not a caregiver to support them on discharge, fact that may also be related to Spanish culture [65]. Regarding to other sociodemographic characteristics, as can be seen in Table 1, the sample was very homogeneous, not showing statistical differences between groups on sex, age, education or marital status, not either in the clinical patients’ data at baseline (stroke type, damaged hemisphere, Oxfordshire Classification and baseline evaluations) which means the participants selected were similar in terms of sequelae left after the stroke, being particularly a group of people who found difficulties in their ADLs but could manage going home (with or without support from family). Regarding the socio-economic status, as can be seen in Table 1, 60% of the participants were retired, therefore, living on retirement pension, 8.3% were househusbands or housewives, 5% were unemployed and only 25% were actively working when the stroke took place, therefore since then being temporarily unfit to work (8 persons in control group and 7 in experimental group). Consequently, it shows there were not significant differences between groups.

Overall, literature addressing OT interventions for stroke survivors going home on discharge shows that, although most of them are effective in terms of patients’ functional recovery, as well as caregivers’ improvements in self-efficacy and fatigue reduction, the heterogeneity of the interventions precludes to draw specific conclusions [11–13]. Stroke discharges and rehabilitation plans are usually carried out in a multidisciplinary manner, making it difficult to evaluate the extent OT contributes to the patients’ recovery [3–5, 54]. Consequently, EOTIPS program was designed and carried out in order to evaluate early OT intervention in the process of hospital discharge and study its influence on the person with stroke quality of life and independence.

As revealed in the Results section, evaluations related to functional independence (mRS), quality of life (SAQOL-39), cognition skills (MOCA) and level of depression (BDI-II) showed better recovery for the experimental group. Quality of life has been shown to be a strong predictor of survival, and this prognostic ability suggests that there is a need for routine assessment of quality of life in clinical trials [66]. Quality of life is a complex concept that is interpreted and defined in a number of ways within and between various disciplines. As a consequence, SAQOL-39 was used in this research due to its validity and reliability within the stroke population [20]. BI may be a useful outcome in stroke rehabilitation RCTs with sufficient sample sizes to support accurate interpretation of statistical significance levels; however, the mRS seems to lack sensitivity to detect change and thus may be unsuitable as a primary outcome [67]. Moreover, while the mRS has a plateau effect in measuring recovery for this patient population between 3 and 6 months, the BI may be a more sensitive measure for assessing recovery up to 12 months [68]. Despite results on mRS scores were positive, Barthel Index did not obtain significant results, therefore, it would be needed a bigger sample size to determine how positive the intervention was on the patient’s independence recovery.

As previously explained, EOTIPS was carried out in parallel to the usual care provided by the healthcare system, in which only a very small number of patients get to see the occupational therapist within the first three months after the stroke due to long waiting lists and reduced number of professionals. In the present RCT, patients with stroke who received EOTIPS showed greater gains in quality of life, independence, perceptual-cognitive skills and levels of depression compared with patients in the usual-care group. The experimental group have shown a 33% higher percentage of change on SAQOL-39 compering to the control group, being consistent with quality-of-life improvements from a previously published study [4]. Nevertheless, Rasmussen et al. [4] intervention group was treated by a multidisciplinary, intersectoral and interventional team, whereas this research only includes the occupational therapist along the conventional care, which includes generally physiotherapy.

Regarding independence, statistically significant results were obtained for mRS but not for BI, although both groups greatly improved its results. Yamakawa et al. [50], referred an active OT intervention on patients with acute stroke was effective in improving the limitations in performing ADLs and reducing the length of hospitalization. They also indicated that it was more effective in patients with severe limitations in performing ADLs and cognitive impairment, such as neglect. Comparing to our study, it is noteworthy the greater improvement of the experimental group on the chosen scale to assess cognition skills, which almost doubled scores. A reason for this result may be the stimulation added by the occupational therapist and caregiver, as well as the better adherence to treatment and compliance of recommended exercises to improve perceptual-cognitive skills [53].

In regards to symptoms of depression, they reduced significantly after intervention in a 69% compared to the control group, being this data cohesive with a previously published study by Feng et al. [5], although they also found a significant decline on levels of anxiety that was not reflected in our research. Nevertheless, different evaluation tools were utilized. No similar studies that include communication skills as a variable have been found.

## Strengths and limitations

This study has several limitations. First of all, one of the limitations may be the selected randomization method which was chosen to simply control balance across groups over time for a sample size of 60 participants. As published data suggest, the 6-block randomization method may not be optimal for randomization, as blocks are best used in smaller increments due to easier balance control. Therefore, the 4-block randomization method could have been a more optimal technique to use for this study [69]. On the other hand, although balance in sample size may be achieved with this method, groups may be generated that are rarely comparable in terms of certain covariates, which could introduce bias in the statistical analysis and reduce the power of the study. Consequently, sample size and covariates should had been balanced in this clinical research [70]. Secondly, although the evaluators were blinded to group assignment, the occupational therapist leading the intervention and who also participated in statistical analysis was not, therefore potential bias may have influenced final outcomes. It should be noted that they had no conflicts of interest. Thirdly, our study included patients with mild-moderate stroke sequelae and our results may not be valid for patients with moderate-severe sequelae. Fourthly, rehabilitation sessions that participants received varied due to many reasons. At the time, the public hospital area where the research took place, provided physiotherapy on ward (see Fig 1), but the number of sessions every patient received depended on the physiotherapist caseload, although it was intended every patient that was referred to physiotherapy by the rehabilitator doctor received at least one session a day. On the other hand, OT was not provided in the ward. In regards to the outpatient rehabilitation, patients could be referred to physiotherapy or OT, but start of intervention varied due to a waiting list, which was especially long for OT, and which meant some participants had not yet been called to start their outpatient’s intervention in the 3 months of research follow-up or were not even referred in the first place. Moreover, data regarding private treatment was inaccessible or not reliable, therefore it has not been included in the results comparisons. Fifthly, not all participants had a main caregiver that could help with their rehabilitation at home, which could also lead to differences on their skills improvements. Frequency and intensity of practice they performed at home also depended on their willingness to improve and adherence to treatment, it being therefore a limitation itself. Finally, participants with moderate-severe aphasia were not included in the investigation.

A previously published RCT by Mudzi et al. [53], concludes reminding the need to devise new ways of providing rehabilitation to patients post discharge enhancing the importance of domiciliary visits, as well as prompts that caregivers require more support to enable them to positively influence patient outcomes post stroke. Similarly, the current research shows a new possibility for the Spanish public healthcare system to include the occupational therapist as an active health professional to maximize safety on discharge and improve stroke patients’ quality of life. An important strength to mention is that, although sample sizes for each group had been previously calculated to prove statistical significance, the results have been shown to be also clinically relevant, revealing better outcomes on quality of life, cognition and depression. Moreover, EOTIPS provides information about the benefits of home assessments and post-discharge support for stroke survivors returning home and their families. Findings can lead to evidence-based clinical practice guideline recommendations, and therefore improvements for patients’ outcomes and greater cost-efficiency in hospitals.

## Conclusions

This study has important implications for Occupational Therapy practice. First, it showed that early OT intervention after stroke can be provided successfully in the Spanish public healthcare system. Secondly, an early OT intervention during the process of discharge can help support participation in daily tasks and activities for patients diagnosed with a stroke, enhancing quality of life and leading to better outcomes on cognition.

This research provides additional support for OT as an evidence-based intervention to improve quality of life, perceptual-cognitive skills, independence and reduce depression in adults with stroke. Further replication studies with larger sample size are needed to validate this intervention.

## Supporting information

S1 File CONSORT checklist. (DOC)

S2 File Study protocol. (PDF)

S3 File Approval by the research ethics committee. (PDF)
