# Supplementary material for: Early Occupational Therapy Intervention post-stroke (EOTIPS): A randomized controlled trial
Source: PLoS One. 2024 Aug 19;19(8):e0308800. doi: 10.1371/journal.pone.0308800 (PMC11332918; doi:10.1371/journal.pone.0308800)
Supplement: S2 File — (PDF) [file pone.0308800.s002.pdf]

Intervención de terapia ocupacional precoz en el proceso de alta hospitalaria tras sufrir un ictus

P García-Pérez[1,2], JP Lara[1], C de la Cruz-Cosme[3], MC Rodríguez-Martínez[2]
[1]Brain Health Unit CIMES-IBIMA-University of Malaga; [2]Health Sciences School-University of Málaga; [3]Neurology Department, Virgen de la Victoria University Hospital of Málaga

RESUMEN

Antecedentes: El ictus constituye la primera causa de discapacidad adquirida en el adulto, siendo una enfermedad cerebrovascular de gran impacto en términos sanitarios y sociales debido, no solo a su prevalencia e incidencia, sino a la gran repercusión que presenta en términos de dependencia y su consecuente impacto en la vida del paciente y su familia.

Objetivos generales y específicos: El objetivo general del presente estudio es determinar si la intervención de terapia ocupacional precoz junto a la atención habitual en el alta hospitalaria tras accidente cerebrovascular tiene un efecto positivo en la calidad de vida del paciente, comparándolo con el grupo control que tendrá la asistencia de cuidados y de rehabilitación habitual. Diseñamos un programa de intervención precoz de TO de cuatro semanas para pacientes que han sufrido un ictus con el objetivo de mejorar su resultado funcional y facilitar la vuelta al hogar aportando conocimientos al paciente o cuidador sobre cuidados específicos y de neurorrehabilitación. Resultado primario: calidad de vida. Resultados secundarios: independencia funcional del paciente y sus necesidades de apoyo en actividades de la vida diaria, destrezas sensitivo-motoras, habilidades perceptivo-cognitivas, habilidades de comunicación y niveles de ansiedad y depresión del paciente. Las evaluaciones se realizarán con todos los pacientes antes del alta y tres meses después.

Diseño del estudio: Este es un ensayo clínico prospectivo, aleatorizado y controlado. El tamaño muestral está conformado por 60 pacientes que estarán divididos en dos grupos: el grupo de control, con 30 usuarios y el grupo experimental, con otros 30 usuarios. La muestra estará compuesta por pacientes que han sufrido un ictus y que han sido dados de alta en el servicio de neurología de un hospital de segundo nivel en Málaga Oeste (España), siendo derivados al servicio de rehabilitación por decisión conjunta del departamento de neurología y rehabilitación. Los pacientes asignados al grupo experimental serán incluidos en un programa de intervención de terapia ocupacional precoz y se compararán con un grupo de control que recibirá la atención y rehabilitación habitual.

Palabras clave: ictus, alta hospitalaria, terapia ocupacional, calidad de vida, cuidador

Antecedentes de la investigación

Llamamos ictus o accidente cerebrovascular (ACV) a aquellos episodios repentinos del aparato circulatorio que comprometen la estabilidad de un área cerebral afectada, ya sea de forma permanente o transitoria. De este modo, usaremos los términos definidos para hacer alusión al infarto cerebral, así como a hemorragias intracerebrales y subaracnoideas (1).

El ictus es una enfermedad cerebrovascular que tiene un gran impacto sanitario y social debido a su elevada incidencia y prevalencia, y constituye la primera causa de discapacidad adquirida en el adulto en países desarrollados. Su incidencia en España es de 187,4/100.000 hab/año. Supone una gran carga, no solo desde el punto de vista sanitario, sino también personal y familiar, por su impacto en la vida de las personas que lo sufren y en la de sus cuidadores (2, 3).

Constituye, además, una de las principales causas de estancia hospitalaria y defunción en nuestro país. La relevancia del ictus viene dada por sus datos de mortalidad, así como de dependencia de los supervivientes. En la fase aguda del ictus, la mortalidad asciende hasta el 25% de los casos, apareciendo más frecuentemente en ictus hemorrágicos (hasta el 50% de fallecimientos) que isquémicos (25%). Los estudios evidencian que en los primeros seis meses tras el evento circulatorio, muchos de estos fallecimientos son causados por complicaciones cardiopulmonares, sin embargo, tras esta etapa se alcanza una estabilización clínica y funcional significativa. Del total de supervivientes, un 30-40% presentará alguna secuela grave, hasta un 60% de usuarios presentará secuelas menores o no tendrá secuelas, y únicamente un 6% de los casos con parálisis inicial grave recuperará completamente la movilidad. Estos datos demuestran la importancia de la rehabilitación para facilitar el reaprendizaje de estos pacientes, habiendo demostrado en estudios previos ser útil en la recuperación del usuario en términos de autonomía funcional, aumentando además la frecuencia de retorno al hogar y reduciendo la estancia hospitalaria. Del mismo modo, se ha demostrado que un programa mediado por el cuidador tiene el potencial de mejorar los resultados de función corporal, actividades y participación de estas personas con ACV. Además de esta forma, los cuidadores participan más activamente en el proceso de rehabilitación, lo que puede aumentar los sentimientos de empoderamiento, reduciendo los niveles de carga del cuidador y facilitando la transición del hospital al hogar (4).

Generalmente, la asistencia inicial en ACV comienza con una llamada a los servicios del 061 o 112 y la llegada a Urgencias. Posteriormente, el usuario es ingresado en la Unidad de Ictus y una vez estabilizado, es trasladado a la planta de neurología. La importancia de la rehabilitación en la fase aguda viene dada por la existencia de una ventana terapéutica durante la cual las intervenciones pueden realmente modificar el curso de la enfermedad y conseguir una reactivación neuronal. Esta mejoría puede deberse a la existencia de un área de penumbra en la periferia de la zona isquémica, cuyo daño es potencialmente reversible, aunque sólo puede conseguirse durante un corto y variable tiempo de hasta 24 horas si se logra la reperfusión de dicho tejido, así como a la resolución de la diasquisis (fallo transináptico de neuronas conectadas con el área dañada). Sin embargo, existe una reorganización cerebral que puede ser modulada por técnicas de rehabilitación a través del fenómeno de plasticidad neuronal, lo que determina la importancia de una eficiente intervención interdisciplinar (4, 5).

El papel del terapeuta ocupacional tiene su lugar en todos los niveles de intervención del ACV, tanto en la actuación directa con el usuario, como en el asesoramiento y en el apoyo familiar o social. La intervención

ocupacional se dirige inicialmente a las habilidades del desempeño sensitivomotoras y perceptivo-cognitivas, así como hacia la reeducación y el entrenamiento en las actividades de la vida diaria (AVD) básicas e instrumentales; posteriormente se plantea una intervención más orientada hacia la integración social y laboral de la persona (6).

La Terapia Ocupacional (TO) interviene sobre la función, empleando procedimientos y actividades específicas para desarrollar, mantener, mejorar y/o recuperar el desempeño de funciones y actividades necesarias en la vida diaria, compensar las disfunciones y/o promover la salud y el bienestar. Según el marco de trabajo para la práctica de terapia ocupacional, clasificamos esta función en áreas de ocupación (AVD básicas e instrumentales, descanso y sueño, educación, trabajo, juego, ocio y participación social) y destrezas de ejecución (destrezas motoras y praxis, destrezas sensoriales-perceptuales, destrezas de regulación emocional, destrezas cognitivas y destrezas sociales y de comunicación). Por lo tanto, el objetivo inicial de un plan de intervención realizado desde TO es mejorar la independencia del usuario en la realización de AVD, consiguiendo el máximo nivel de autonomía posible y promoviendo su bienestar y calidad de vida (6, 7).

De acuerdo con un estudio publicado en 2015, el pronóstico del ACV en España ha cambiado en los últimos años. Tanto la supervivencia como el resultado funcional han mejorado como resultado de la introducción de un nuevo modelo de atención. Este nuevo modelo, basado en unidades de ictus, las terapias de reperfusión precoz, fisioterapia y prevención secundaria, ha sido promovido por la estrategia de accidente cerebrovascular del Servicio Nacional de Salud de España en coordinación con las autoridades de salud regionales y los profesionales de la salud. Sin embargo, este modelo no tiene en cuenta la intervención de TO en el proceso de alta, como sí hacen otros países con resultados positivos en la independencia funcional, entre otros (8).

Debe recordarse por último que esta patología también tiene un impacto económico relevante en el costo directo sanitario nacional, más los que se deriven de su repercusión laboral y socio-familiar. Según un estudio publicado recientemente, el coste de los pacientes ingresados en unidades de ictus en España es de 27.711 € por paciente / año. Más de dos tercios son costos sociales, principalmente atención informal (9).

Fundamentación

El modelo de alta temprana con apoyo o *early supported discharge* (ESD) para rehabilitación se introdujo a finales de la década de 1990 e incluye un equipo interdisciplinario con los recursos adecuados que coordina el alta y planifica, supervisa y continúa la rehabilitación en el entorno del hogar (10).

En base a ensayos controlados aleatorizados realizados, existe evidencia de que el alta temprana con seguimiento del hospital (ESD) y con rehabilitación continua en el hogar tiene efectos beneficiosos en los usuarios de ictus; sin embargo, no se han investigado los efectos del servicio de ESD en la práctica clínica habitual en España (11, 12). Del mismo modo, se evidencia una escasez de datos publicados en cuanto a la repercusión de la Terapia Ocupacional en el proceso de alta hospitalaria junto a la atención y rehabilitación convencional en términos de resultados funcionales del paciente, carga del cuidador y costos de la atención médica durante los primeros seis meses tras el ACV (13).

Es importante tener en cuenta la implicación de la familia en el proceso de

rehabilitación, así como sus conocimientos con respecto a la enfermedad y cuidados del paciente para proporcionar un entorno estimulante y seguro en el hogar que reduzca riesgos de úlceras, acortamientos musculares o de tejidos blandos, luxaciones, etc., en pacientes con déficit motor secuelar. El tratamiento que se presta en la sanidad pública actual es insuficiente lo que puede ser preocupante debido a la limitada recuperación posterior al accidente cerebrovascular y la dificultad en la reintegración comunitaria. Es por ello que ciertas variables son de especial interés al tratar de ponderar la eficacia de este tipo de actuaciones, como es la mejoría motora y la importancia de la movilidad funcional de las extremidades superiores, que pueden contribuir a una mejor calidad de vida para los supervivientes de ictus (13, 14).

Hay al menos tres perspectivas importantes a tener en cuenta en cualquier proyecto de valoración de las actuaciones sobre las secuelas de ACV: lo personal, lo social y la salud pública. Desde la perspectiva personal, volver a casa tras una estancia hospitalaria por ictus y poder participar en la planificación de la rehabilitación, así como decidir los objetivos tiene como resultado el empoderamiento del paciente y una mayor satisfacción, mejorando el estado de ánimo y promoviendo el bienestar. Desde la perspectiva social, contribuye a que la familia se sienta menos angustiada cuando se brinda apoyo en el hogar, reduce el riesgo de reingreso y los síntomas inducidos por el estrés en los familiares, lo cual es también una oportunidad para mejorar la calidad de vida del cuidador y reducir su riesgo de enfermedad ya que los familiares a menudo informan sentir una gran carga como cuidadores. Desde la perspectiva de la salud pública, la posibilidad de que sea seguro dar de alta con el apoyo de terapia ocupacional significa una disminución de costes. No hay estudios en nuestro entorno que demuestren si el alta con apoyo de TO es eficaz y rentable, si incrementa la seguridad de pacientes y familiares, o si los pacientes perciben menos ansiedad y depresión, mayores niveles de independencia funcional y una mejor calidad de vida (5).

Objetivos de investigación

Objetivo general

El objetivo general del presente estudio es determinar si la intervención precoz de terapia ocupacional (TO) junto a la atención habitual en el proceso de alta hospitalaria tras ACV tiene un efecto positivo en la calidad de vida del paciente, comparándolo con el grupo control que tendrá la asistencia de cuidados y de rehabilitación habitual.

Diseñamos un programa de intervención precoz de TO de cuatro semanas para pacientes que han sufrido un ictus con el objetivo de mejorar su calidad de vida y facilitar la vuelta al hogar aportando conocimientos al paciente y al cuidador sobre cuidados específicos y de neurorrehabilitación.

Objetivos específicos

Resultado primario: calidad de vida. Resultados secundarios: independencia funcional y necesidades de apoyo en actividades de la vida diaria, destrezas sensitivo-motoras, habilidades perceptivo-cognitivas, habilidades de comunicación y niveles de ansiedad y depresión.

Metodología

Tipo y diseño general del estudio

Este es un ensayo clínico prospectivo, aleatorizado y controlado. La población a representar es la de los pacientes que son dados de alta en el servicio de neurología del Hospital Universitario Virgen de la Victoria (Málaga), y que, tras el alta médica, son derivados al servicio de rehabilitación del Hospital Marítimo de Torremolinos por decisión de los departamentos de neurología y rehabilitación como pacientes

ambulatorios, pero que no serán subsidiarios de una cama en la Unidad de Medicina Física y Rehabilitación para rehabilitación intensiva.

Los usuarios que se derivan a rehabilitación obteniendo plaza como paciente interno, reciben terapia ocupacional y fisioterapia diariamente durante un periodo de máximo tres meses. Sin embargo, los pacientes que se derivan al servicio de rehabilitación como paciente externo, son trasladados a casa directamente tras el alta y son posteriormente llamados para realizar la rehabilitación. El periodo que trascurre desde que son dados de alta hasta que son llamados para comenzar su rehabilitación varía según la carga de trabajo del servicio, pudiendo ser incluso de tres meses tras el alta. La derivación a rehabilitación externa o intensiva se realiza en base al potencial de rehabilitación, que tiene en cuenta determinados criterios como la edad, la situación clínica y la lesión vascular existente en el momento del alta, el apoyo social, la motivación y la recuperación observada durante el ingreso. Los pacientes asignados al programa de estudio son incluidos en un programa de intervención de terapia ocupacional precoz y se comparan con un grupo de control que recibe la atención y rehabilitación habitual.

Selección y tamaño de la muestra

El tamaño muestral "n" se calculó en base a la variable principal SAQOL-39 con la fórmula de determinación de tamaño muestral para dos grupos independientes, asumiendo una media de 2.50, desviación típica de 0.75, nivel de significación del 5%, una potencia del 80% y una diferencia crítica de 0.54, obteniendo una muestra de 30 participantes por grupo.

Por lo tanto, se conformarán dos grupos: un grupo de intervención de 30 pacientes que recibirá el programa de Terapia Ocupacional precoz (EOTIPS) además de la rehabilitación y atención convencional, y un grupo control de 30 pacientes que recibirán la rehabilitación y atención habitual.
Los pacientes elegibles fueron asignados a EOTIPS o al grupo de control según una designación preestablecida por ordenador para asegurar una aleatorización por bloques de seis.

Criterios de inclusión y exclusión

Todos los participantes deben tener confirmación diagnóstica de ictus con lesiones vasculares únicas o múltiples que hubieran ocurrido en el mismo periodo temporal, demostradas por pruebas de neuroimagen (TAC o RMN), ser mayores de 18 años de edad, volver a su domicilio al alta y que esta se encuentre a máximo 30 minutos de distancia del centro hospitalario, con >2 ó <26 puntos en la escala del Instituto Nacional de Salud (NIHSS), 30–100 puntos en el Índice de Barthel (BI) al segundo día del ictus (con BI 100, el paciente puede incluirse si la Evaluación Cognitiva de Montreal es <26) y derivación al servicio de rehabilitación como paciente ambulatorio. La inclusión al estudio se produce de forma previa al alta hospitalaria, en un intervalo de dos semanas tras el padecimiento del ictus.

Los criterios de exclusión son: NIHSS> 26, BI <30, y esperanza de vida <1 año. Se excluyen también las personas con ACV previo, demencia u otro tipo de enfermedades que cursen con demencia y otras enfermedades neurológicas, psiquiátricas o médicas (por ejemplo, epilepsia grave, traumatismo craneoencefálico, esquizofrenia, EPOC, cardiopatía grave o inestable, apnea del sueño) concomitantes que pudieran alterar la función cognitiva, presencia de afasia moderada o severa, personas que no comprenden español o inglés, así como pacientes que han sido derivados a la Unidad de Medicina Física y Rehabilitación como paciente encamado para recibir rehabilitación intensiva o que son ingresados en residencia.

En las variables se tendrán en cuenta: edad, sexo, nivel de estudios, estado civil, nacionalidad, lengua materna, empleo, territorio vascular y volumen de la lesión.

Relación de resultados y evaluaciones

Evaluaciones del paciente	
Calidad de vida	- SAQOL-39
Nivel de independencia funcional	- BI<br>- mRS<br>- SIS-16
Habilidades perceptivo-cognitivas	- MoCA
Destrezas sensitivo-motoras	- FMA<br>- BBS<br>- TUG
Habilidad para comunicarse	- CAL
Niveles de ansiedad y/o depresión	- BDI-II<br>- HAM-A

Evaluaciones a utilizar en el estudio (todas traducidas al español)

- *Escala de calidad de vida de accidente cerebrovascular y afasia - 39, SAQOL-39* (Lata-Caneda, Piñeiro-Temprano, García-Fraga, García-Armesto, Barrueco-Egido, Meijide-Failde, 2009). El SAQOL-39 se derivó de la Escala de calidad de vida específica del accidente cerebrovascular y de cuatro elementos adicionales específicamente dirigidos a pacientes con afasia, que abarca cuatro dominios: físico, psicosocial, comunicación y energía.

- *Evaluación Cognitiva de Montreal*, *MoCA* (Lozano Gallego, Hernández Ferrándiz, Turró Garriga, Pericot Nierga, López-Pousa, Vilalta Franch, 2009). El MoCA se ha propuesto como una herramienta de detección que promete una buena sensibilidad a los déficits que derivan del accidente cerebrovascular y el deterioro cognitivo vascular. El MoCA incluye secciones sobre funciones visuoespaciales/ ejecutivas, nomenclatura, atención, lenguaje, abstracción, memoria y orientación. Se puntúa sobre 30 (punto extra para <13 años de educación) y el límite "normal" recomendado es ≥26.

- *Modified Rankin Scale*, *mRS* (Van Swieten, Kodustaal, Visser, Schouten, Van Gijn, 1988). El mRS se utiliza para describir la discapacidad en general. La escala va de 0 a 6, desde una salud perfecta sin síntomas hasta la muerte.

- *Fugl-Meyer sensory motor assessment, FMA* (Ferrer González, Zarco Periñán, Ruiz de Vargas, Docobo Durantez, 2015). Evalúa la extremidad superior (puntuación máxima de 66 que corresponde a la función motora normal) y extremidad inferior (puntuación máxima de 34). También se evalúan la sensibilidad, el rango de movimiento pasivo y el dolor durante los movimientos pasivos de las articulaciones.

- *Timed up and go, TUG* (Hafsteinsdóttir et al, 2014). El TUG evalúa la movilidad básica, cronometrando el tiempo requerido para que una persona se levante de una silla estandarizada, camine una distancia de tres metros, gire, regrese a la silla y vuelva a sentarse. Un tiempo más corto indica un mejor rendimiento. Se ha demostrado que es confiable y válido en este grupo de pacientes.

- *Berg Balance Scale, BBS* (Kudlac , Sabol, Kaiser, Kane, Phillips, 2019). El BBS evalúa el equilibrio funcional. El rendimiento en esta prueba se califica de 0 (no se puede realizar) a 4 (rendimiento normal) en 14 tareas diferentes, incluida la capacidad de sentarse, pararse, alcanzar, inclinarse, darse la vuelta y dar un paso. El puntaje máximo en el BBS es 56. Un puntaje más alto indica mejores habilidades de equilibrio.

- *Índice de Barthel, BI* (Baztán, Pérez del Molino, Alarcón, San Cristobal, Izquierdo, Manzarbeitia, 1993). El índice Barthel mide hasta qué punto alguien puede funcionar de forma independiente durante las actividades básicas de la vida diaria (AVD), es decir, alimentación, baño, aseo, vestimenta, control intestinal, control de la vejiga, aseo, traslado de silla, deambulación y subir escaleras. Cada elemento de rendimiento se clasifica en esta escala con un número dado de puntos asignados a cada nivel. En este estudio se utiliza la versión modificada con 0–100, donde una puntuación más baja indica dependencia.

- E*scala de Impacto del Ictus, Stroke Impact Scale - 16, SIS-16* (Duncan, Lai, Bode, Perera, DeRosa, 2003) . La utilidad de esta escala es similar a la del Índice de Barthel, aunque es más sensible que éste último para discriminar entre pacientes con discapacidades leves. Está diseñada especialmente para pacientes que han sufrido ictus, pero es aplicable también en demencias. Es una versión reducida del Stroke Impact Scale versión 3.0. La información puede obtenerse del mismo paciente, o del cuidador principal (15) (16).

- *Communicative Activity Log, CAL* (Pulvermüller & Berthier, 2008). Escala que permite obtener información de la capacidad de comunicación en las actividades de la vida diaria referida a aspectos comprensivos y expresivos del lenguaje. Está compuesta por 36 ítems (escala tipo Likert, 0-6) que evalúan tanto la calidad (ítems 1-18) como la cantidad de comunicación del paciente (ítems 19-36). Se puede aplicar de forma autoadministrada o heteroadministrada. El rendimiento se obtiene a través de la suma de las puntuaciones en cada ítem.

- *Inventario de depresión de Beck, BDI-II* (Steer, Brown, 1996). Es un cuestionario autoadministrado que consta de 21 preguntas de respuesta múltiple. Es uno de los instrumentos más comúnmente utilizados para medir la severidad de una depresión.

- *Escala de ansiedad de Hamilton, HAM-A* (Lobo et al, 2002). Esta escala evalúa la severidad de la ansiedad de una forma global en pacientes que reúnan criterios de ansiedad o depresión. Además, este instrumento es útil para monitorizar la respuesta al tratamiento. Está compuesto por 14 ítems, siendo 13 referentes a signos y síntomas ansiosos y el último que valora el comportamiento del paciente durante la entrevista.

Las evaluaciones se realizarán en ambos grupos (control y experimental) antes del alta (tras cumplimentación del consentimiento informado) y tres meses después del evento neurológico.

Cronograma y recolección de datos

Planificación del alta en el hospital

Los departamentos de rehabilitación y neurología serán los encargados de derivar los pacientes al terapeuta ocupacional e incluirlos en el estudio. Antes de comenzar la rehabilitación, el terapeuta ocupacional y sus familiares exploran las necesidades y deseos del paciente y deciden los objetivos individuales para el período de intervención.

Intervención de Terapia Ocupacional durante el proceso de alta

La intervención tiene un enfoque centrado en la persona que se basa en quién es el usuario: su contexto, su historia, sus familiares, sus fortalezas y debilidades individuales. Se establecerán objetivos individualizados, teniendo en cuenta las potencialidades y limitaciones del paciente. Ejemplos de objetivos pueden ser: poder asearse independientemente, colgar la ropa o viajar en autobús o cómo administrar las facturas. La intervención de TO puede

implicar la capacitación en diferentes actividades o pensar en diferentes formas de adaptarse a situaciones difíciles para lograr una meta. Por ejemplo, para algunos pacientes la intervención puede consistir en realizar las AVD en las que presentan una limitación con el apoyo del terapeuta ocupacional con el objetivo de potenciar su sentido de seguridad durante el desempeño de la misma. Los datos se recopilarán mediante toma de notas y se analizarán con análisis de contenido cualitativos.

La intervención de TO diseñada comprende:

- Previo al alta: Evaluación inicial y una sesión en el hospital.
- Posterior al alta: Una visita en el hogar de dos horas, seguimiento telefónico, una visita de una hora al mes del alta y evaluación final a los tres meses.

Esta intervención de TO precoz se llevará a cabo independientemente de la atención habitual del servicio de rehabilitación del Hospital Marítimo de Torremolinos.

Planificación temporal

El proyecto comenzará en enero de 2021 y se estima que durará un año y tres meses (finalización estimada en marzo de 2022).

Día 1: Hospital (1 hora)<br>Entrevista inicial y evaluación.
Día 2: Hospital (1 hora)<br>Información sobre el ictus. Cuidados posturales.
Día 3: Vivienda (2 horas)<br>Estudio del entorno, recomendaciones y productos de apoyo y establecer objetivos individualizados.
Día 4 - día 29: SEGUIMIENTO TELEFÓNICO. Puesta en marcha de la rehabilitación por parte del paciente y el cuidador (objetivos individuales).
Día 30: Vivienda (1 hora).<br>Valoración final de objetivos individuales en el hogar.
A los 3 meses: Vivienda (1 hora)<br>Evaluación final.

Plan de análisis de los resultados

Se seguirá el siguiente modelo de análisis: para estudiar las diferencias que puede haber entre un grupo y otro, antes y después de la intervención, se realizará una ANOVA de medidas repetidas; para comprobar cómo influyen unas variables con respecto a otras se puede realizar un análisis multivariante y, por último, para conocer la media y la distribución normal de las variables se realizará una estadística descriptiva. Se incluirá un análisis intragrupo realizando estudios pareados de T-test junto con Wilcolxon para medidas no paramétricas. El programa a utilizar para el análisis de datos será JAMOVI (versión 2.3.26).

Dificultades y limitaciones del estudio

En cuanto a las limitaciones de este estudio, cabe destacar los sesgos posiblemente derivados de la adherencia al tratamiento de los usuarios y sus cuidadores, así como la conducta e implicación del familiar en el proceso que de forma probable inducirá a diferencias en los resultados. Cabe destacar la importancia de la palabra del paciente y del cuidador debido al proceso telefónico de la intervención, ya que puede dar lugar a diferencias en las instrucciones aportadas por el terapeuta ocupacional y, por lo tanto, en el tratamiento y resultado final.

Además, una importante limitación es la heterogeneidad de la muestra, pues hablamos en general de pacientes que hayan tenido un ictus de cualquier localización, extensión y volumen. La distribución aleatoria de los pacientes puede tener como consecuencia que los dos grupos tengan diferencias significativas importantes por la gravedad de sus lesiones o por su potencial de recuperación. Debido también a la heterogeneidad del proceso de rehabilitación de cada paciente, se entiende que, del tamaño muestral total, determinados pacientes recibirán posteriormente un mayor número de

sesiones de rehabilitación convencional que otros, e incluso probablemente algún paciente del grupo control no reciba ninguna sesión. Es por ello que parece interesante estudiar posteriormente la atención recibida por cada usuario a los tres meses del alta, así como su nivel de funcionalidad y calidad de vida.

Consideraciones éticas

Durante la recogida de datos se garantizará el anonimato de los usuarios. Se informará a los participantes de la intervención y se les pedirá la firma de un consentimiento informado, siendo la participación voluntaria y permitiendo que el usuario abandone cuando lo desee.

Se tendrán en cuenta los principios de la declaración de Helsinki (17).

Se pedirá la autorización al hospital, así como la aprobación del comité ético de investigación provincial al que el centro está adscrito.

Referencias bibliográficas

1. 2017-Guia-Prevencion-Ictus.pdf [Internet]. [citado 10 de noviembre de 2020]. Disponible en: https://www.portalfarma.com/Profesionales/campanaspf/categorias/Documents/2017-Guia-Prevencion-Ictus.pdf
2. Informe_ICTUS_Andalucia.pdf [Internet]. [citado 10 de noviembre de 2020]. Disponible en: https://www.sen.es/images/2020/atlas/Informes_comunidad/Informe_ICTUS_Andalucia.pdf
3. Abordaje del accidente cerebrovascular.pdf [Internet]. [citado 24 de marzo de 2020]. Disponible en: https://www.mscbs.gob.es/biblioPublic/publicaciones/docs/200204_1.pdf
4. Vloothuis JD, Mulder M, Veerbeek JM, Konijnenbelt M, Visser-Meily JM, Ket JC, et al. Caregiver-mediated exercises for improving outcomes after stroke. Cochrane Stroke Group, editor. Cochrane Database Syst Rev [Internet]. 21 de diciembre de 2016 [citado 27 de marzo de 2020]; Disponible en: http://doi.wiley.com/10.1002/14651858.CD011058.pub2
5. Cuadrado ÁA. Rehabilitación del ACV: evaluación, pronóstico y tratamiento. Galicia Clínica. 2009;70(3):25-40.
6. Mercado Padín, R. RR A. MARCO DE TRABAJO PARA LA PRÁCTICA DE TERAPIA OCUPACIONAL: Dominio y Proceso 2da Edición. 2010.
7. Ros YD, Bouhsain ME, Salcedo IV, Cantín RC. Plan of intervention from occupational therapy in a patient with right hemiplegia: treatment reoutfitter centre don functionality of upper extremity. :9.
8. on behalf of CONOCES Investigators Group, Mar J, Masjuan J, Oliva-Moreno J, Gonzalez-Rojas N, Becerra V, et al. Outcomes measured by mortality rates, quality of life and degree of autonomy in the first year in stroke units in Spain. Health Qual Life Outcomes. diciembre de 2015;13(1):36.
9. Alvarez-Sabín J, Quintana M, Masjuan J, Oliva-Moreno J, Mar J, Gonzalez-Rojas N, et al. Economic impact of patients admitted to stroke units in Spain. Eur J Health Econ HEPAC Health Econ Prev Care. mayo de 2017;18(4):449-58.
10. Bråndal A, Eriksson M, Glader E-L, Wester P. Effect of early supported discharge after stroke on patient reported outcome based on the Swedish Riksstroke registry. BMC Neurol. 12 de marzo de 2019;19(1):40.
11. Langhorne P, Baylan S, Trialists ESD. Early supported discharge services for people with acute stroke. Cochrane Database Syst Rev [Internet]. 2017 [citado 10 de noviembre de 2020];(7). Disponible en: https://www.cochranelibrary.com/cdsr/doi/10.1002/14651858.CD000443.pub4/full
12. Fearon P, Langhorne P, Trialists ESD. Services for reducing duration of hospital care for acute stroke patients. Cochrane Database Syst Rev [Internet]. 2012 [citado 17 de noviembre de 2020];(9). Disponible en: https://www.cochranelibrary.com/cdsr/doi/10.1002/14651858.CD000443.pub3/full
13. García AMD. OCCUPATIONAL THERAPY'S TREATMENT IN CEREBROVASCULAR DAMAGES. Mª. :24.
14. Lieshout ECC van, van de Port IG, Dijkhuizen RM, Visser-Meily JMA. Does upper limb strength play a prominent role in health-related quality of life in stroke patients discharged from inpatient rehabilitation? Top Stroke Rehabil. 9 de marzo de 2020;1-9.
15. Carod-Artal FJ. Escalas específicas para la evaluación de la calidad de vida en el ictus. REV NEUROL. :11.
16. Reeves M, Lisabeth L, Williams L, Katzan I, Kapral M, Deutsch A, et al. Patient-Reported Outcome Measures (PROMs) for Acute Stroke: Rationale, Methods and Future Directions. Stroke. 2018;49(6):1549-56.
17. WMA - The World Medical Association-WMA Declaration of Helsinki – Ethical Principles for Medical Research Involving Human Subjects [Internet]. [citado 10 de noviembre de 2020]. Disponible en: https://www.wma.net/policies-post/wma-declaration-of-helsinki-ethical-principles-for-medical-research-involving-human-subjects/

STUDY PROTOCOL

Early occupational therapy intervention in the hospital discharge process after suffering a stroke

P García-Pérez[1,2], JP Lara[1], C de la Cruz-Cosme[3], MC Rodríguez-Martínez[2]
[1]Brain Health Unit CIMES-IBIMA-University of Malaga; [2]Health Sciences School-University of Málaga; [3]Neurology Department, Virgen de la Victoria University Hospital of Málaga

ABSTRACT

Background: Stroke is the first cause of acquired disability in adults, being a cerebrovascular disease with great impact in health and social terms due, not only to its prevalence and incidence, but also to the great impact it has in terms of dependency and its consequent impact on the life of the patient and their family.

General and specific objectives: The general objective of the present study is to determine if the intervention of early occupational therapy together with usual care at hospital discharge after stroke has a positive effect on the quality of life of the patient, comparing it with the control group that will have regular care and rehabilitation. We designed a four-week early Occupational Therapy (OT) intervention program in patients who have suffered a stroke with the aim of improving their functional outcome and facilitating their return home by providing the patient and caregiver with knowledge about specific care and neurorehabilitation. Primary outcome: functional independence of the patient and their support needs in activities of daily living. Secondary outcomes: improvement in sensory-motor skills, perceptual-cognitive skills, communication skills, quality of life, and patient anxiety and depression levels. Assessments will be conducted with all patients before discharge and three months later.

Study design: This is a prospective, randomized, controlled clinical trial. The sample size is made up of 60 patients who will be divided into two groups: the control group, with 30 users and the experimental group, with another 30 users. The sample will be made up of patients who have suffered a stroke and who have been discharged from the neurology service of a second level hospital in West Malaga (Spain), being referred to the rehabilitation service by joint decision of the neurology department and rehabilitation. Patients assigned to the experimental group will be included in an early occupational therapy intervention program and will be compared with a control group that will receive usual care and rehabilitation.

Keywords: stroke, hospital discharge, occupational therapy, quality of life, caregiver

Research background

We call a stroke or cerebrovascular accident (CVA) those sudden episodes of the circulatory system that compromise the stability of an affected brain area, whether permanently or temporarily. In this way, we will use the defined terms to refer to cerebral infarction, as well as intracerebral and subarachnoid hemorrhages (1).

Stroke is a cerebrovascular disease that has a great health and social impact due to its high incidence and prevalence, and is the leading cause of acquired disability in adults in developed countries. Its incidence in Spain is 187.4/100,000 inhabitants/year. It represents a great burden, not only from a health point of view, but also from a personal and family point of view, due to its impact on the lives of people who suffer from it and their caregivers (2, 3).

It is also one of the main causes of hospital stay and death in our country. The relevance of stroke is given by its mortality data, as well as the dependency of survivors. In the acute phase of stroke, mortality rises to 25% of cases, appearing more frequently in hemorrhagic strokes (up to 50% of deaths) than ischemic strokes (25%). Studies show that in the first six months after the circulatory event, many of these deaths are caused by cardiopulmonary complications; however, after this stage, significant clinical and functional stabilization is achieved. Of the total survivors, 30-40% will present some serious sequelae, up to 60% of users will present minor sequelae or no sequelae, and only 6% of cases with severe initial paralysis will completely recover mobility. These data demonstrate the importance of rehabilitation to facilitate the relearning of these patients, having been shown in previous studies to be useful in the user's recovery in terms of functional autonomy, also increasing the frequency of return home and reducing hospital stay. Similarly, a caregiver-mediated program has been shown to have the potential to improve bodily function, activity, and participation outcomes for these individuals with stroke. In addition, caregivers participate more actively in the rehabilitation process, which can increase feelings of empowerment, reducing caregiver burden levels and easing the transition from hospital to home (4).

Generally, initial assistance in stroke begins with a call to the 061 or 112 services and arrival at the Emergency Department. Subsequently, the user is admitted to the Stroke Unit and once stabilized, they are transferred to the neurology ward. The importance of rehabilitation in the acute phase is given by the existence of a therapeutic window during which interventions can truly modify the course of the disease and achieve neuronal reactivation. This improvement may be due to the existence of an area of penumbra in the periphery of the ischemic zone, whose damage is potentially reversible, although it can only be achieved for a short and variable time of up to 24 hours if reperfusion of said tissue is achieved, as well as the resolution of diaschisis (transsynaptic failure of neurons connected to the damaged area). However, there is a brain reorganization that can be modulated by rehabilitation techniques through the phenomenon of neuronal plasticity, which determines the importance of an efficient interdisciplinary intervention (4, 5).

The role of the occupational therapist has its place at all levels of stroke intervention, both in direct action with the user, as well as in counseling and family or social support. Occupational intervention is initially directed at sensorimotor and perceptual-cognitive performance skills, as well as re-education and training in basic and instrumental activities of daily living (ADL); Subsequently, an intervention more oriented towards the social and labor integration of the person is proposed (6).

Occupational Therapy (OT) intervenes on function, using specific procedures and activities to develop, maintain, improve and/or recover the performance of functions

and activities necessary in daily life, compensate for dysfunctions and/or promote health and well-being. Based on the framework for occupational therapy practice, we classify this function into areas of occupation (basic and instrumental ADLs, rest and sleep, education, work, play, leisure, and social participation) and performance skills (motor skills and praxis, sensory-perceptual skills, emotional regulation skills, cognitive skills, and social and communication skills). Therefore, the initial objective of an intervention plan carried out from OT is to improve the user's independence in carrying out ADLs, achieving the highest possible level of autonomy and promoting their well-being and quality of life (6, 7).

According to a study published in 2015, the prognosis of stroke in Spain has changed in recent years. Both survival and functional outcome have improved as a result of the introduction of a new model of care. This new model, based on stroke units, early reperfusion therapies, physiotherapy and secondary prevention, has been promoted by the stroke strategy of the Spanish National Health Service in coordination with regional health authorities and healthcare professionals. health. However, this model does not take into account the intervention of OT in the discharge process, as other countries with positive results in functional independence do, among others (8).

Finally, it should be remembered that this pathology also has a relevant economic impact on the direct national health cost, plus those derived from its work and socio-family repercussions. According to a recently published study, the cost of patients admitted to stroke units in Spain is €27,711 per patient/year. More than two-thirds are social costs, mainly informal care (9).

Foundation

The early supported discharge (ESD) model for rehabilitation was introduced in the late 1990s and includes a well-resourced interdisciplinary team that coordinates discharge and plans, monitors, and continues rehabilitation in the hospital setting. home (10).

Based on randomized controlled trials conducted, there is evidence that early discharge with hospital follow-up (ESD) and with continued rehabilitation at home has beneficial effects in stroke users; However, the effects of the ESD service in routine clinical practice in Spain have not been investigated (11, 12). Likewise, there is a lack of published data regarding the impact of Occupational Therapy in the hospital discharge process along with conventional care and rehabilitation in terms of patient functional outcomes, caregiver burden, and health care costs. during the first six months after the stroke (13).

It is important to take into account the involvement of the family in the rehabilitation process, as well as their knowledge regarding the patient's illness and care to provide a stimulating and safe environment at home that reduces the risk of ulcers, muscle or tissue shortening. soft joints, dislocations, etc., in patients with sequelae motor deficit. The treatment provided in current public healthcare is insufficient, which may be worrying due to the limited recovery after stroke and the difficulty in community reintegration. This is why certain variables are of special interest when trying to assess the effectiveness of this type of action, such as motor improvement and the importance of functional mobility of the upper extremities, which can contribute to a better quality of life for patients. stroke survivors (13, 14).

There are at least three important perspectives to take into account in any project to assess actions on the consequences of stroke: the personal, the social and public health. From a personal

perspective, returning home after a hospital stay for stroke and being able to participate in rehabilitation planning, as well as deciding goals, results in patient empowerment and greater satisfaction, improving mood and promoting well-being. From a social perspective, it helps the family feel less distressed when support is provided at home, it reduces the risk of readmission and stress-induced symptoms in family members, which is also an opportunity to improve the caregiver quality of life and reduce their risk of illness, as family members often report feeling a great burden as caregivers. From a public health perspective, making it safe to discharge with occupational therapy support means decreased costs. There are no studies in our environment that demonstrate whether discharge with OT support is effective and profitable, whether it increases the safety of patients and families, or whether patients perceive less anxiety and depression, higher levels of functional independence and a better quality of life. (5).

Research objectives

General Objective

The general objective of the present study is to determine if the early intervention of occupational therapy (OT) together with usual care in the hospital discharge process after stroke has a positive effect on the quality of functional independence of the patient, comparing it with the control group that will have the assistance regular care and rehabilitation.

We designed a four-week early OT intervention program for patients who have suffered a stroke with the aim of improving quality of life and facilitating their return home by providing them and their caregiver with knowledge about specific care and neurorehabilitation.

Specific Objectives

Primary outcome: Quality of life. Secondary results: Functional independence and support needs in activities of daily living, sensory-motor skills, perceptual-cognitive skills, communication skills, anxiety and depression levels.

Methods

Type and general design of the study

This is a prospective, randomized, controlled clinical trial. The population to be represented is that of patients who are discharged from the neurology service of the Virgen de la Victoria University Hospital (Malaga) and who, after medical discharge, are referred to the rehabilitation service of the Maritime Hospital of Torremolinos as outpatients by decision of the neurology and rehabilitation departments, but will not be inpatients in the Physical Medicine and Rehabilitation Unit for intensive rehabilitation.

Users who are referred to rehabilitation by obtaining a place as an inpatient, receive occupational therapy and physiotherapy daily for a period of maximum three months. However, patients who are referred to the rehabilitation service as an outpatient are taken home directly after discharge and are subsequently recalled for rehabilitation. The period that elapses from when they are discharged until they are called to begin their rehabilitation varies depending on the workload of the service, and can even be three months after discharge. Referral to external or intensive rehabilitation is made based on the rehabilitation potential, which takes into account certain criteria such as age, clinical situation and vascular injury existing at the time of discharge, social support, motivation and observed recovery. during admission. Patients assigned to the study program are included in an early occupational therapy intervention program and are compared with a control group that will receive usual care and rehabilitation.

Sample selection and size

The sample size "n" was calculated based on the main variable SAQOL-39 with the sample size determination formula for two independent groups, assuming a mean of 2.50, standard deviation of 0.75, significance level of 5%, a power of 80% and a critical difference of 0.54, obtaining a sample of 30 participants per group.

Therefore, two groups will be formed: an intervention group of 30 patients who will receive the early Occupational Therapy program in addition to the rehabilitation that they would receive independently of this study, and a control group of 30 patients who will receive the conventional rehabilitation.
Eligible patients were assigned to EOTIPS or the control group according to a pre-established computer randomized designation in blocks of six.

Inclusion and exclusion criteria

All patients must have diagnostic confirmation of stroke with single or multiple vascular lesions that had occurred in the same time period, demonstrated by neuroimaging tests (CT or MRI), be over 18 years of age, go home on discharge and live within a maximum of 30 minutes away from the hospital center, with >2 or <26 points on the National Institute of Health scale (NIHSS), 30–100 points on the Barthel Index (BI) on the second day after the stroke (with BI 100, the patient can be included if the Montreal Cognitive Assessment is <26) and having been referred to the rehabilitation service as an outpatient. Inclusion in the study occurs prior to hospital discharge, within a two-week interval after the stroke.

Exclusion criteria are: NIHSS > 26, BI < 30, and life expectancy < 1 year. Also excluded are people with a previous stroke, dementia or other types of diseases that cause dementia and other neurological, psychiatric or medical diseases (for example, severe epilepsy, traumatic brain injury, schizophrenia, COPD, severe or unstable heart disease, sleep apnea) concomitants that could alter cognitive function, presence of moderate or severe aphasia, people who do not understand Spanish or English, as well as patients who have been referred to the Physical Medicine and Rehabilitation Unit as inpatients to receive intensive rehabilitation or who are discharged to a care home.

The variables will take into account: age, sex, educational level, marital status, nationality, mother tongue, employment, vascular territory and lesion volume.

List of results and evaluations

Patient evaluations	
Quality of life	- SAQOL-39
Level of functional independence	- BI<br>- mRS<br>- SIS-16
Perceptual-cognitive skills	- MoCA
Sensory-motor skills	- Fugl Meyer<br>- Berg<br>- Time up & go
Communication skills	- CAL
Anxiety and/or depression levels	- Berg<br>- Hamilton

Assessments to be used in the study (all translated into Spanish)

- *Stroke and Aphasia Quality of Life Scale - 39, SAQOL-39* (Lata-Caneda, Piñeiro-Temprano, García-Fraga, García-Armesto, Barrueco-Egido, Meijide-Failde, 2009). SAQOL-39 was derived from the Stroke-Specific Quality of Life Scale and four additional items specifically targeting patients with aphasia, covering four domains: physical, psychosocial, communication, and energy.

- *Montreal Cognitive Assessment*, *MoCA* (Lozano Gallego, Hernández Ferrándiz,

Turró Garriga, Pericot Nierga, López-Pousa, Vilalta Franch, 2009). MoCA has been proposed as a screening tool that promises good sensitivity to deficits resulting from stroke and vascular cognitive impairment. MoCA includes sections on visuospatial/executive functions, naming, attention, language, abstraction, memory, and orientation. It is scored out of 30 (extra point for <13 years of education) and the recommended "normal" limit is ≥26.

- *Modified Rankin Scale*, *mRS* (Van Swieten, Kodustaal, Visser, Schouten, Van Gijn, 1988). The mRS is used to describe disability in general. The scale ranges from 0 to 6, from perfect health with no symptoms to death.

- *Fugl-Meyer sensory motor assessment, FMA* (Ferrer González, Zarco Periñán, Ruiz de Vargas, Docobo Durantez, 2015). It evaluates the upper extremity (maximum score of 66 which corresponds to normal motor function) and lower extremity (maximum score of 34). Tenderness, passive range of motion, and pain during passive joint movements are also evaluated.

- *Timed up and go, TUG* (Hafsteinsdóttir et al, 2014). TUG assesses basic mobility, timing the time required for a person to stand up from a standardized chair, walk a distance of three meters, turn, return to the chair, and sit down again. A shorter time indicates better performance. It has been shown to be reliable and valid in this group of patients.

- *Berg Balance Scale, BBS* (Kudlac, Sabol, Kaiser, Kane, Phillips, 2019). BBS assesses functional balance. Performance on this test is scored from 0 (unable to perform) to 4 (normal performance) on 14 different tasks, including the ability to sit, stand, reach, bend, turn, and step. The maximum score on the BBS is 56. A higher score indicates better balance skills.

- *Barthel Index, BI* (Baztán, Pérez del Molino, Alarcón, San Cristobal, Izquierdo, Manzarbeitia, 1993). The Barthel Index measures the extent to which someone can function independently during basic activities of daily living (ADLs), i.e., feeding, bathing, grooming, dressing, bowel control, bladder control, grooming, chair transfers, ambulation and climbing stairs. Each performance element is rated on this scale with a given number of points assigned to each level. In this study, the modified version with 0–100 is used, where a lower score indicates dependence.

- *Stroke Impact Scale - 16, SIS-16* (Duncan, Lai, Bode, Perera, DeRosa, 2003). The usefulness of this scale is similar to that of the Barthel Index, although it is more sensitive than the latter for discriminating between patients with mild disabilities. It is designed especially for patients who have suffered a stroke, but it is also applicable to dementia. It is a reduced version of the Stroke Impact Scale version 3.0. The information can be obtained from the patient himself, or from the main caregiver (15) (16).

- *Communicative Activity Log, CAL* (Pulvermüller & Berthier, 2008). Scale that allows obtaining information on the communication capacity in the activities of daily life referring to comprehensive and expressive aspects of language. It is composed of 36 items (Likert-type scale, 0-6) that evaluate both the quality (items 1-18) and the quantity of patient communication (items 19-36). It can be applied self-administered or heteroadministered. Performance is obtained through the sum of the scores on each item.

- *Beck Depression Inventory, BDI-2* (Steer, Brown, 1996). It is a self-administered questionnaire that consists of 21 multiple-choice questions. It is one

of the most commonly used instruments to measure the severity of depression.

- *Hamilton Anxiety Scale* (Lobo et al, 2002). This scale evaluates the severity of anxiety globally in patients who meet criteria for anxiety or depression. In addition, this instrument is useful to monitor the response to treatment. It is composed of 14 items, 13 referring to anxious signs and symptoms and the last one assessing the patient's behavior during the interview.

The evaluations will be carried out in both groups (control and intervention group) before discharge (after completion of the informed consent) and three months after the neurological event.

Timeline and data collection

Hospital discharge planning

The rehabilitation and neurology departments will be responsible for referring patients to the occupational therapist and including them in the study. Before starting rehabilitation, the occupational therapist and her family explore the patient's needs and desires and decide individual goals for the intervention period.

Occupational Therapy intervention during the discharge process
The intervention has a person-centered approach that is based on who the user is: their context, their history, their family members, their individual strengths and weaknesses. Individualized objectives will be established, taking into account the patient's potential and limitations. Examples of goals could be: being able to wash independently, hanging clothes or riding the bus, or how to manage bills. OT intervention may involve training in different activities or thinking about different ways to adapt to difficult situations to achieve a goal. For example, for some patients the intervention may consist of carrying out ADLs in which they have a limitation with the support of the occupational therapist with the aim of enhancing their sense of security during its performance. Data will be collected through note taking and analyzed with qualitative content analysis.

The designed OT intervention includes:
- Prior to discharge: Initial evaluation and a hospital session.
- Post-discharge: A two-hour home visit, telephone follow-up, a one-hour visit one month after discharge and final evaluation three months later.

This early OT intervention will be carried out independently of the usual care of the rehabilitation service of the Torremolinos Maritime Hospital.

Time planning
The project will begin in January 2021 and is estimated to last one year and three months (estimated completion in March 2022).

Day 1: Hospital (1 hour)<br>Initial interview and evaluation.
Day 2: Hospital (1 hour)<br>Information about stroke. Postural care.
Day 3: Home (2 hours)<br>Environment assessment, equipment and recommendations and establishment of individualized objectives.
Day 4 - day 29: PHONE FOLLOW-UP. Implementation of the rehabilitation by the patient and caregiver (individual objectives).
Day 30: Home (1 hours).<br>Final assessment of individualized objectives at home.
After 3 months: Home (1 hour)<br>Final evaluation.

Results analysis plan
The following analysis model will be followed: to study the differences that may exist between one group and another, before and after the intervention, a repeated measures ANOVA will be carried out; to check how some variables influence others, a multivariate analysis can be carried out and, finally, to know the mean and normal distribution of the variables,

descriptive statistics will be performed. An intragroup analysis will be included by performing paired T-test studies along with Wilcolxon for non-parametric measurements. The program to be used for data analysis will be JAMOVI (version 2.3.26).

Difficulties and limitations of the study

Regarding the limitations of this study, it is worth highlighting the biases possibly derived from the adherence to treatment of the users and their caregivers, as well as the behavior and involvement of the family member in the process that will likely induce differences in the results. It is worth highlighting the importance of the word of the patient and the caregiver due to the telephone process of the intervention, since it can lead to differences in the instructions provided by the occupational therapist and, therefore, in the treatment and final result.

Furthermore, an important limitation is the heterogeneity of the sample, since we are generally talking about patients who have had a stroke of any location, extent and volume. The random distribution of patients may result in the two groups having significant differences in the severity of their injuries or their potential for recovery. Also due to the heterogeneity of each patient's rehabilitation process, it is understood that, of the total sample size, certain patients will subsequently receive a greater number of conventional rehabilitation sessions than others, and some patients will probably not even receive any sessions. This is why it seems interesting to subsequently study the care received by each user three months after discharge, as well as their level of functionality and quality of life.

Ethical considerations

During data collection, the anonymity of users will be guaranteed. Participants will be informed of the intervention and will be asked to sign an informed consent, with participation being voluntary and allowing the user to abandon whenever they wish.

The principles of the Declaration of Helsinki (17) will be taken into account.
Authorization will be requested from the hospital, as well as approval from the provincial research ethics committee to which the center is attached.

References

1. 2017-Guia-Prevencion-Ictus.pdf [Internet]. [citado 10 de noviembre de 2020]. Disponible en: https://www.portalfarma.com/Profesionales/campanaspf/categorias/Documents/2017-Guia-Prevencion-Ictus.pdf
2. Informe_ICTUS_Andalucia.pdf [Internet]. [citado 10 de noviembre de 2020]. Disponible en: https://www.sen.es/images/2020/atlas/Informes_comunidad/Informe_ICTUS_Andalucia.pdf
3. Abordaje del accidente cerebrovascular.pdf [Internet]. [citado 24 de marzo de 2020]. Disponible en: https://www.mscbs.gob.es/biblioPublic/publicaciones/docs/200204_1.pdf
4. Vloothuis JD, Mulder M, Veerbeek JM, Konijnenbelt M, Visser-Meily JM, Ket JC, et al. Caregiver-mediated exercises for improving outcomes after stroke. Cochrane Stroke Group, editor. Cochrane Database Syst Rev [Internet]. 21 de diciembre de 2016 [citado 27 de marzo de 2020]; Disponible en: http://doi.wiley.com/10.1002/14651858.CD011058.pub2
5. Cuadrado ÁA. Rehabilitación del ACV: evaluación, pronóstico y tratamiento. Galicia Clínica. 2009;70(3):25-40.
6. Mercado Padín, R. RR A. MARCO DE TRABAJO PARA LA PRÁCTICA DE TERAPIA OCUPACIONAL: Dominio y Proceso 2da Edición. 2010.
7. Ros YD, Bouhsain ME, Salcedo IV, Cantín RC. Plan of intervention from occupational therapy in a patient with right hemiplegia: treatment reoutfitter centre don functionality of upper extremity. :9.
8. on behalf of CONOCES Investigators Group, Mar J, Masjuan J, Oliva-Moreno J, Gonzalez-Rojas N, Becerra V, et al. Outcomes measured by mortality rates, quality of life and degree of autonomy in the first year in stroke units in Spain. Health Qual Life Outcomes. diciembre de 2015;13(1):36.
9. Alvarez-Sabín J, Quintana M, Masjuan J, Oliva-Moreno J, Mar J, Gonzalez-Rojas N, et al. Economic impact of patients admitted to stroke units in Spain. Eur J Health Econ HEPAC Health Econ Prev Care. mayo de 2017;18(4):449-58.
10. Bråndal A, Eriksson M, Glader E-L,

Wester P. Effect of early supported discharge after stroke on patient reported outcome based on the Swedish Riksstroke registry. BMC Neurol. 12 de marzo de 2019;19(1):40.
11. Langhorne P, Baylan S, Trialists ESD. Early supported discharge services for people with acute stroke. Cochrane Database Syst Rev [Internet]. 2017 [citado 10 de noviembre de 2020];(7). Disponible en: https://www.cochranelibrary.com/cdsr/doi/10.1002/14651858.CD000443.pub4/full
12. Fearon P, Langhorne P, Trialists ESD. Services for reducing duration of hospital care for acute stroke patients. Cochrane Database Syst Rev [Internet]. 2012 [citado 17 de noviembre de 2020];(9). Disponible en: https://www.cochranelibrary.com/cdsr/doi/10.1002/14651858.CD000443.pub3/full
13. García AMD. OCCUPATIONAL THERAPY'S TREATMENT IN CEREBROVASCULAR DAMAGES. Mª. :24.
14. Lieshout ECC van, van de Port IG, Dijkhuizen RM, Visser-Meily JMA. Does upper limb strength play a prominent role in health-related quality of life in stroke patients discharged from inpatient rehabilitation? Top Stroke Rehabil. 9 de marzo de 2020;1-9.
15. Carod-Artal FJ. Escalas específicas para la evaluación de la calidad de vida en el ictus. REV NEUROL. :11.
16. Reeves M, Lisabeth L, Williams L, Katzan I, Kapral M, Deutsch A, et al. Patient-Reported Outcome Measures (PROMs) for Acute Stroke: Rationale, Methods and Future Directions. Stroke. 2018;49(6):1549-56.
17. WMA - The World Medical Association-WMA Declaration of Helsinki – Ethical Principles for Medical Research Involving Human Subjects [Internet]. [citado 10 de noviembre de 2020]. Disponible en: https://www.wma.net/policies-post/wma-declaration-of-helsinki-ethical-principles-for-medical-research-involving-human-subjects/
