# Supplementary material for: Early Occupational Therapy Intervention post-stroke (EOTIPS): A randomized controlled trial
Source: PLoS One. 2024 Aug 19;19(8):e0308800. doi: 10.1371/journal.pone.0308800 (PMC11332918; doi:10.1371/journal.pone.0308800)
Supplement: S3 File — (PDF) [file pone.0308800.s003.pdf]

DOCUMENTO ESTADO DE PROYECTO

Título completo:	Intervención de terapia ocupacional precoz en el proceso de alta hospitalaria tras sufrir un ictus.
Código del estudio:	02/21PI13
Promotor:	(No hay promotor/a asociado/a)
Comité:	CEI de Centro Provincial de Málaga
Protocolo:	PROTOCOLO ESTUDIO TERAPIA OCUPACIONAL ICTUS.pdf
Versión Protocolo:	1.0
Fecha Protocolo:	28/12/2021
HIP:	HOJA DE INFORMACIO?N A PACIENTE.pdf
Versión HIP:	1.0
Fecha HIP:	28/12/2021
Solicitante:	CARLOS DE LA CRUZ COSME
NIF solicitante:	74851681K
Fecha actual:	13/02/2021
Estado:	ENVIADO

Centros del proyecto

Investigador/a principal	Centros participantes	Servicio
CARLOS DE LA CRUZ COSME	HOSPITAL UNIVERSITARIO VIRGEN DE LA VICTORIA	
JOSE PABLO LARA MUÑOZ	Universidad de Málaga	
MARIA DEL CARMEN RODRIGUEZ MARTINEZ	Universidad de Málaga	
PATRICIA GARCIA PEREZ	LAS LAGUNAS	

Documentos del proyecto

Nombre	Version	Fecha
IDONEIDAD INVESTIGADORES TERAPIA OCUPACIONAL.pdf	1.0	
COMPROMISO INVESTIGADORES TERAPIA OCUPACIONAL_compressed.pdf	1.0	
IDONEIDAD INSTALACIONES TERAPIA OCUPACIONAL.pdf	1.0	
PROTOCOLO ESTUDIO TERAPIA OCUPACIONAL ICTUS.pdf	1.0	
HOJA DE INFORMACIO?N A PACIENTE.pdf	1.0	
CONSENTIMIENTO INFORMADO .pdf	1.0	

Estados del proyecto

Estado final	Fecha
PENDIENTE DE ENVÍO	11/02/2021
ENVIADO	13/02/2021

Dra. Dña. Gloria Luque Fernández, Secretaria del CEI Provincial de Málaga

CERTICA:

Que en la sesión de CEI de fecha: 25/02/2021 ha evaluado la propuesta de D/Dña.: Carlos de la Cruz Cosme, referido al Proyecto de Investigación: "Intervención de terapia ocupacional precoz en el proceso de alta hospitalaria tras sufrir un ictus".

Este Comité lo considera ética y metodológicamente correcto.

La composición del CEI en esta sesión es la siguiente:

Dra. Ana Alonso Torres (UGC Neurociencias)
Dra. Encarncación Blanco Reina (Farmacología Clínica)
Dra. Begoña Jiménez Rodríguez (UGC Oncología)
Dra. Marta Blasco Alonso (Obst. y Ginecología)
Dr. Rafael Carvia Ponsaille (Anatomía Patológica)
Dª. Ana Díaz Ruíz (Licenciada en Derecho)
Dr. José C. Fernández García (UGC Endocrinología y Nutrición)
Dr. Manuel Herrera Gutiérrez (UGC UCI)
Dra. Begoña Jiménez Rodríguez (UGC Oncología)
Dr. José Leiva Fernández (Médico Familia)
Dra. Mª Dolores López Carmona (Medicina Interna)
Dr. Jesús López del Peral (Esp.Protec.Datos)
Dña. Carmen López Gálvez del Postigo (Miembro Lego)
Dr. Antonio López Téllez (Médico de Familia)
Dra. Gloria Luque Fernández (Investigación)
Dra. Cristobalina Mayorga Mayorga (Laboratorio)
Dra. Mª Angeles Rosado Souvirón (UGC Farmacia)
Dra. Leonor Ruíz Sicilia (UGC Salud Mental)
Dra. Mª Victoria de la Torre Prados (UMA)
Dª. Inmaculada Doña Díaz (Alergología)
Dr. Andrés Fontalba Navas (UGC Salud Mental)
D. José Manuel García Cabello
Dr. Victor Navas López (UGC Pediatría)
Dra. Mª Carmen Vela Márquez (Farmacéutica Distrito)

Lo que firmo en Málaga, a 3 de marzo de 2021

Fdo.: Dra. Gloria Luque Fernández
Secretaria del CEI

PROJECT STATUS DOCUMENT

Full title: Early occupational therapy intervention in the hospital discharge process after suffering a stroke.

Study code: 02/21PI13

Promoter: (There is no associated promoter)

Committee: CEI of the Provincial Center of Malaga (Provincial Research Ethics Committee of Malaga).

Protocol: OCCUPATIONAL THERAPY STROKE STUDY PROTOCOL.pdf

Protocol Version: 1.0

Protocol Date: 28/12/2021

HIP: PATIENT INFORMATION SHEET.pdf

HIP version: 1.0

HIP Date: 28/12/2021

Applicant: CARLOS DE LA CRUZ COSME

Applicant NIF: 74851681K

Current date: 13/02/2021

State: SENT

Project centers

**Principal researcher**	**Participating centers**	**Service**
CARLOS DE LA CRUZ COSME	VIRGEN DE LA VICTORIA UNIVERSITY HOSPITAL	
JOSE PABLO LARA MUÑOZ	UNIVERSITY OF MALAGA	
MARIA DEL CARMEN RODRIGUEZ MARTINEZ	UNIVERSITY OF MALAGA	
PATRICIA GARCIA PEREZ	LAS LAGUNAS	

Project documents

**Name**	**Version**	**Date**
SUITABILITY OF OCCUPATIONAL THERAPY RESEARCHERS.pdf	1.0	
RESEARCHER COMMITMENT OCCUPATIONAL THERAPY_compressed.pdf	1.0	
SUITABILITY OF OCCUPATIONAL THERAPY FACILITIES.pdf	1.0	
OCCUPATIONAL THERAPY STROKE STUDY PROTOCOL.pdf	1.0	
PATIENT INFORMATION SHEET.pdf	1.0	
INFORMED CONSENT .pdf	1.0	

Project status

**Final status**	**Date**
SENDING PENDING	11/02/2021
SENT	13/02/2021

Provincial Research Ethics Committee of Málaga (CEI)

Dr. Gloria Luque Fernández, Secretary of the provincial CEI of Málaga

CERTIFIES

That during the CEI session that took place on 25th February 2021, it was evaluated the proposal of Mr. Carlos de la Cruz Cosme regarding the Research Project entitled: "Early Occupational Therapy Intervention in the process of hospital discharge after stroke".

This committee considers the mentioned project is ethically and methodologically correct.

The composition of this CEI is the following:

Dra. Ana Alonso Torres (Neurosciences UGC)
Dra. Encarnación Blanco Reina (Clinical Pharmacology)
Dra. Begoña Jiménez Rodríguez (Oncology UGC)
Dra. Marta Blasco Alonso (Obstetrics and Gynecology)
Dr. Rafael Carvia Ponsaille (Pathology Anatomy)
Ms. Ana Díaz Ruíz (Graduate in Law)
Mr. José C. Fernández García (Endocrinology and Nutrition UGC)
Dr. Manuel Herrera Gutiérrez (ICU UGC)
Dra. Begoña Jiménez Rodríguez (Oncology UGC)
Dr. José Leiva Fernández (Family Doctor)
Dra. Mª Dolores López Carmona (Internal Medicine)
Dr. Jesús López del Peral (Data Protection Specialist)
Ms. Carmen López Gálvez del Postigo (Non-Expert Member)
Dr. Antonio López Téllez (Family Doctor)
Dra. Gloria Luque Fernández (Research)
Dra. Cristobalina Mayorga Mayorga (Laboratory)
Dra. Mª Angeles Rosado Souvirón (Pharmacy UGC)
Dra. Leonor Ruíz Sicilia (Mental Health UGC)
Dra. Mª Victoria de la Torre Prados (UMA)
Ms. Inmaculada Doña Díaz (Allergology)
Dr. Andrés Fontalba Navas (Mental Health UGC)
Mr. José Manuel García Cabello
Dr. Víctor Navas López (Pediatrics UGC)
Dra. Mª Carmen Vela Márquez (District Pharmacist)

Which I sign in Málaga, 3rd March 2021

Signature: Dra. Gloria Luque Fernández
CEI Secretary

* UGC = Clinical Management Unit
* UMA = University of Málaga
